# Tuning Transcription Factor Availability through Acetylation-Mediated Genomic Redistribution

**DOI:** 10.1016/j.molcel.2020.05.025

**Published:** 2020-08-06

**Authors:** Pakavarin Louphrasitthiphol, Robert Siddaway, Alessia Loffreda, Vivian Pogenberg, Hans Friedrichsen, Alexander Schepsky, Zhiqiang Zeng, Min Lu, Thomas Strub, Rasmus Freter, Richard Lisle, Eda Suer, Benjamin Thomas, Benjamin Schuster-Böckler, Panagis Filippakopoulos, Mark Middleton, Xin Lu, E. Elizabeth Patton, Irwin Davidson, Jean-Philippe Lambert, Matthias Wilmanns, Eiríkur Steingrímsson, Davide Mazza, Colin R. Goding

**Affiliations:** 1 Ludwig Institute for Cancer Research, Nuffield Department of Clinical Medicine, University of Oxford, Headington, Oxford OX3 7DQ, UK; 2 Department of Gastrointestinal and Hepato-Biliary-Pancreatic Surgery, Faculty of Medicine, University of Tsukuba, 1-1-1 Tennodai, Tsukuba, Ibaraki 305-8575, Japan; 3 Experimental Imaging Center, Cancer Imaging Unit, IRCCS San Raffaele Scientific Institute, Via Olgettina 58, 20132 Milan, Italy; 4 Fondazione CEN, European Center for Nanomedicine, 20133 Milan, Italy; 5 European Molecular Biology Laboratory, Hamburg Unit, Notkestrasse 25a, 22607 Hamburg, Germany & University Hamburg Medical Centre Hamburg-Eppendorf, Martinistrasse 52, 20246 Hamburg, Germany; 6 Department of Biochemistry and Molecular Biology, Faculty of Medicine, University of Iceland, Sturlugata 8, 101 Reykjavik, Iceland; 7 MRC Institute of Genetics and Molecular Medicine, MRC Human Genetics Unit and Edinburgh Cancer Research UK Centre, Crewe Road South, Edinburgh EH4 2XR, UK; 8 Institut de Génetique et Biologie Moléculaire et Cellulaire (IGBMC), Equipe labéllisée Ligue contre le Cancer, 1 rue Laurent Fries, 67404 Illkirch Cedex, France; 9 Central Proteomics Facility, Sir William Dunn Pathology School, Oxford University, Oxford OX1 3RE, UK; 10 Ludwig Institute for Cancer Research, Big Data Institute, University of Oxford, Headington, Oxford OX3 7LF, UK; 11 Structural Genomics Consortium, Nuffield Department of Clinical Medicine, University of Oxford, Headington, Oxford OX3 7DQ, UK; 12 Oxford NIHR Biomedical Research Centre, Department of Oncology, Churchill Hospital, Oxford OX3 7LE, UK; 13 Department of Molecular Medicine and Cancer Research Centre, Université Laval, Quebec, QC, Canada; 14 CHU de Québec Research Center, CHUL, 2705 Boulevard Laurier, Quebec G1V 4G2, QC, Canada

**Keywords:** acetylation, E-box, DNA-binding affinity, MITF, bHLH-LZ, melanoma, transcription factor, melanocyte

## Abstract

It is widely assumed that decreasing transcription factor DNA-binding affinity reduces transcription initiation by diminishing occupancy of sequence-specific regulatory elements. However, *in vivo* transcription factors find their binding sites while confronted with a large excess of low-affinity degenerate motifs. Here, using the melanoma lineage survival oncogene MITF as a model, we show that low-affinity binding sites act as a competitive reservoir *in vivo* from which transcription factors are released by mitogen-activated protein kinase (MAPK)-stimulated acetylation to promote increased occupancy of their regulatory elements. Consequently, a low-DNA-binding-affinity acetylation-mimetic MITF mutation supports melanocyte development and drives tumorigenesis, whereas a high-affinity non-acetylatable mutant does not. The results reveal a paradoxical acetylation-mediated molecular clutch that tunes transcription factor availability via genome-wide redistribution and couples BRAF to tumorigenesis. Our results further suggest that p300/CREB-binding protein-mediated transcription factor acetylation may represent a common mechanism to control transcription factor availability.

## Highlights

• Reducing transcription factor DNA-binding affinity increases activity *in vivo*
• Acetylation is triggered by MAPK signaling
• Acetylation leads to genome-wide transcription factor redistribution
• Acetylation of MITF drives tumorigenesis and melanocyte development

## Graphical Abstract

**Figure:**
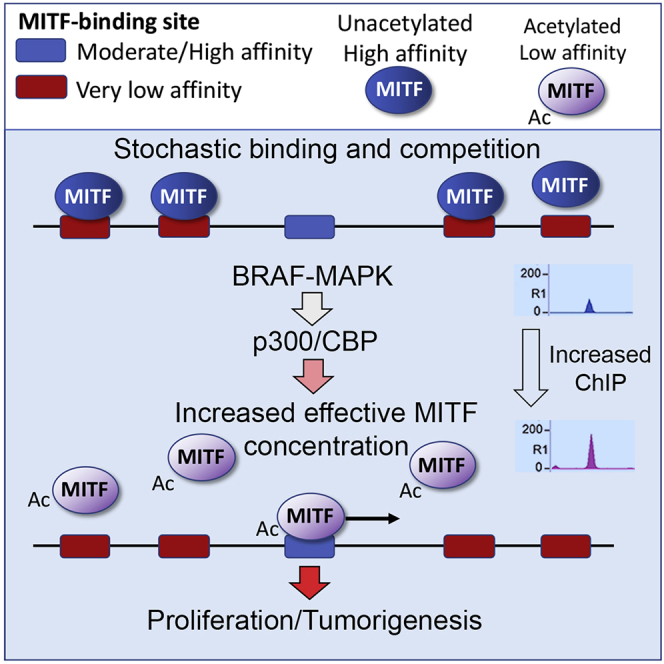

## Teaser

Decreasing the DNA-binding affinity of a transcription factor via MAPK-stimulated p300/CBP-mediated acetylation of a lysine contacting the DNA-phosphate backbone paradoxically increases its functional output by promoting release from a genome-wide reservoir of low-affinity binding sites.

## Introduction

Transcription factors interpret and integrate the output from signal transduction pathways to impose gene expression programs that underpin development and homeostasis, while their deregulation drives cancer progression. The recognition of specific DNA sequence elements in promoter or enhancer regions determines which genes are regulated by individual transcription factors. The repertoire of sites available is restricted by nucleosomes, but for the binding motifs that are accessible, occupancy is determined by a combination of abundance and affinity of the transcription factor for DNA, combined with the affinity of the binding site for the transcription factor. Increasing the dwell time of a transcription factor on a DNA-recognition element will lead to increased transcription (Lickwar et al., 2012). Consequently, it is widely assumed that decreasing the affinity of a transcription factor for DNA will impair its capacity for gene regulation. However, for each sequence-specific regulatory element, the genome contains many more degenerate variants that could potentially act as a competitive reservoir to titrate transcription factor availability. In principle, regulating a transcription factor’s DNA-binding affinity could facilitate exchange between such a reservoir and its regulatory elements. Whether mammalian cells use regulated genome-mediated titration to tune the effective concentration of a transcription factor is not known.

The wide-ranging biological functions and defined target elements of the basic-helix-loop-helix-leucine zipper (bHLH-LZ) microphthalmia-associated transcription factor MITF (Hodgkinson et al., 1993) in melanoma and melanocyte biology make it an excellent model for understanding transcription factor dynamics in mammalian cells. MITF (Goding and Arnheiter, 2019), a lineage survival oncogene (Garraway et al., 2005), coordinates differentiation, regulates proliferation, suppresses migration/invasiveness and tumor-initiation capacity, and controls lysosome biogenesis, autophagy, and drug sensitivity (Carreira et al., 2005, Carreira et al., 2006, Cheli et al., 2011, Dugo et al., 2015, Hoek et al., 2008, Konieczkowski et al., 2014, Müller et al., 2014, Ploper et al., 2015, Zhang et al., 2015, Möller et al., 2019). It also suppresses senescence (Giuliano et al., 2010). Like other bHLH or bHLH-LZ family members, MITF recognizes 6-bp E-box motifs, with flanking sequences permitting MITF and other E-box-binding factors to discriminate between related target sites (Aksan and Goding, 1998, Blackwell and Weintraub, 1990, Fisher et al., 1993, Fisher and Goding, 1992, Hejna et al., 2019, Solomon et al., 1993). Despite the key role of MITF in melanoma progression, surprisingly little is known about how its target gene selectivity is modulated. Although mitogen-activated protein kinase (MAPK) signaling regulates MITF nuclear export (Ngeow et al., 2018) and enhances interaction with the transcription cofactor CREB-binding protein (CBP)/p300 (Price et al., 1998), how the deregulated BRAF or NRAS signaling that drives melanoma progression otherwise affects MITF’s capacity to regulate gene expression is poorly understood.

Here, we reveal that paradoxically, BRAF-activated p300-mediated acetylation of a transcription factor can increase its function *in vivo* by decreasing its DNA-binding affinity.

## Results

### Genome-wide Distribution of MITF

**Figure 1:**
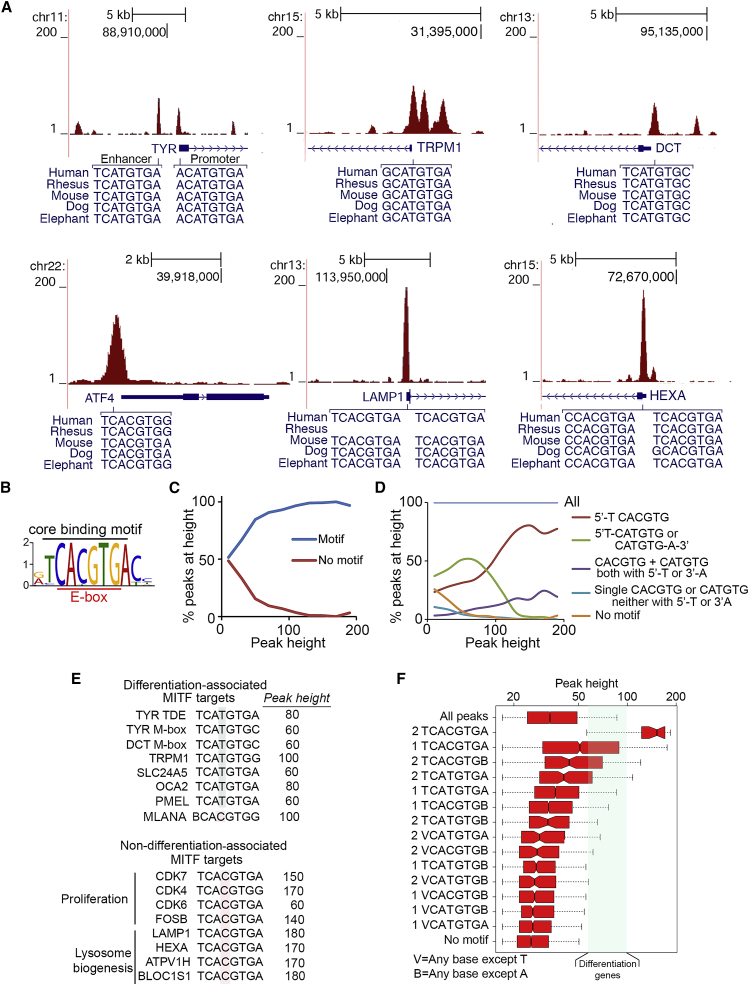
Genome-wide Binding by MITF (A) Genome browser screenshots derived from ChIP-seq using anti-HA antibody of 501mel cells stably expressing ectopic HA-tagged MITF. (B) Consensus motif for the most significant 900 genome-wide MITF-binding sites predicted from 60-bp regions around peak summits generated by MEME. (C) The proportion of peaks with or without a 5′-TCA(T/C)GTGN-3′ motif at different peak heights. (D) Relationship between motif frequency and peak height as in (C). (E) Sequences associated with a selection of differentiation or non-differentiation-associated MITF target genes. (F) Box and whisker plots of peak height related to motif. Center of notches indicates the median. Green box indicates range of peak heights within which lie a set of well-characterized differentiation-associated genes in addition to many other non-differentiation genes. See also Figure S1 and Table S1.

Differentiation-associated MITF target genes possess a conserved M-box (Lowings et al., 1992) comprising a CATGTG E-box motif flanked by 5′T and/or 3′A residues (Aksan and Goding, 1998). By contrast, several MITF targets apparently have E-box elements lacking the 5′T and 3′A flanking residues including *MET*, *KIT*, *BCL2*, *HPGDS*, and *TPSB2* (Cheli et al., 2010). How MITF might discriminate between these binding sites is not known. To address this issue, we performed chromatin immunoprecipitation coupled to high-throughput DNA sequencing (ChIP-seq) using an established (Laurette et al., 2015) 501mel melanoma cell line expressing hemagglutinin (HA)-epitope-tagged MITF (Figures S1A and S1B, upper panel). The results confirmed binding to known MITF target genes, including differentiation genes containing conserved CATGTG motifs such as *TYR* (Figure 1A; Figure S1B, lower panel); *TRPM1* and *DCT* (Figure 1A); *ATF4*, a mediator of the integrated stress response; and *LAMP1* and *HEXA*, lysosomal biogenesis genes that contain CACGTG-binding motifs. In each case, the core 6-bp binding motif was flanked by 5′T and/or 3′A residues that differentiate between MITF and MYC targets (Aksan and Goding, 1998, Fisher et al., 1993, Fisher and Goding, 1992, Hejna et al., 2019, Solomon et al., 1993). No binding was observed at the mast-cell-specific *HPGDS* and *TPSB2* loci. At the *MET*, *KIT*, or *BCL2* genes, MITF recognized intronic motifs (Figure S1C), rather than their promoters as reported previously (McGill et al., 2002, McGill et al., 2006, Tsujimura et al., 1996). Thus, MITF-binding sites contain motifs with a 5′T and/or 3′A and flanking sequences do not distinguish between targets associated with MITF’s different biological functions such as proliferation or differentiation. Consistent with this, the consensus motif for MITF recognition was TCACGTGA (Figure 1B), reflecting that CACGTG motifs are enriched among the highest affinity binding sites.

MITF ChIP peak heights varied between genes (Figure 1A) and the proportion of peaks with binding motifs increased with peak height (Figure 1C). Of the peaks called, high peak heights were associated with CACGTG elements with the flanking T-residue characteristic of MITF binding, whereas CATGTG motifs related to the differentiation-associated M-box sequence exhibited lower peak heights, consistent with a lower affinity for MITF (Figure 1D). As reported previously (Goding, 2000), differentiation-associated genes, with the exception of *MLANA*, which contains a CACGTG element, possess core CATGTG motifs and exhibit peak heights between 60 and 100 (Figure 1E). By contrast, many other MITF targets, frequently associated with proliferation or lysosome biogenesis genes (Ploper et al., 2015, Zhang et al., 2015), had substantially higher peak heights (Figure 1E). Analyzing peak height distribution relative to motif beneath the peak (Figures 1F and S1D) revealed that the highest ChIP peaks (median height, 152) possessed double MITF 8-bp binding sites and the next-highest possessed single TCACGTGA motifs (median height, 51). The more than additive peak heights associated with double binding sites suggest that MITF dimers may bind cooperatively. Thus, peak heights tend to diminish with variations from the consensus 8-bp TCACGTGA element, indicating that for most sites, peak height reflects the binding motif. Note that the median peak height corresponded well to the MITF-bound sequence (Figure 1F), but each motif was associated with a considerable range, most likely reflecting the position of each recognition element relative to nucleosomes or binding sites for cooperating transcription factors.

Although TCATGTGA elements bound MITF less well than TCACGTGA motifs (Figure 1F), the range of peak heights for differentiation genes (60–100) (Figure 1E; Table S1) was similar to the top range of TCACGTGA elements. It was also among the very highest for TCATGTGA motifs in general (Figure 1F); of 23,500 peaks with single TCATGTGA motifs bound by MITF, almost 19,000 exhibited peaks lower than the bottom end of the range, and only 0.2% had peak heights above. Since peak height may reflect affinity for a particular element, DNA-binding affinity does not distinguish between well-characterized differentiation-associated MITF targets and non-differentiation MITF targets.

To eliminate the possibility that the expression of HA-tagged MITF and the endogenous protein could produce different results, we also re-analyzed a ChIP-seq dataset obtained from melanocytes using an anti-MITF antibody (Webster et al., 2014). The results were similar with differences likely accounted for by the reduced number of peaks in the Webster et al. dataset compared to ours, reflecting the high ChIP efficiency when using the anti-HA antibody. Thus, the consensus MITF recognition motif is similar (Figure S1E), mean peak height tends to reduce when the binding site varies from the consensus (Figure S1F) and binding to genes such as *ATF4* and *LAMP1* is located in a similar position (Figure S1G).

### MITF Is Acetylated on a Highly Conserved Phosphate-Backbone-Interacting Lysine

DNA-binding affinity is dictated by a combination of base-specific contacts that provide sequence specificity and contacts with the phosphate backbone. We hypothesized that as MITF-occupied sites exhibit a wide range of peak heights, the relative distribution of MITF binding between low- and high-affinity targets might be regulated through alteration of MITF’s DNA-binding affinity.

**Figure 2:**
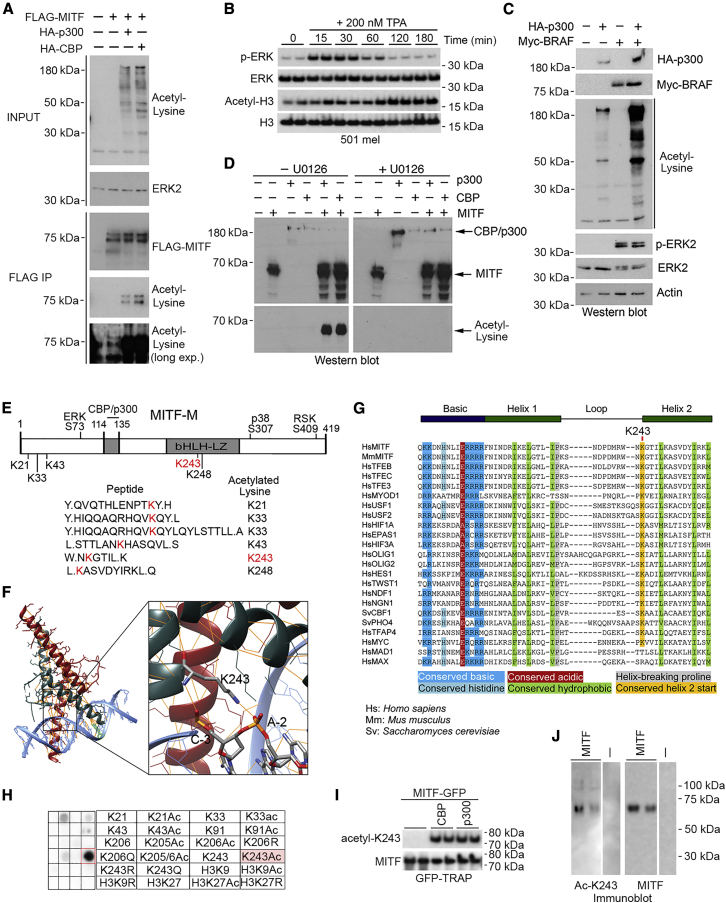
MITF Can Be Acetylated (A) Indicated expression vectors were transfected into Phoenix cells and input and anti-FLAG immunoprecipitates western blotted. (B) Western blot of 501mel cells treated with 200 nM TPA for indicated times. (C) Western blot of extracts from cells transfected with BRAF and/or p300 expression vectors. (D) Western blot of Phoenix cells transfected with indicated vectors and HA-MITF, ±20 μM U0126 immunoprecipitated using anti-HA antibody. (E) Schematic showing the melanocyte-specific MITF-M(+) isoform. The five acetylated lysine residues identified in MITF-M peptides by mass spectrometry are indicated below. ERK, p38, and RSK phosphorylation sites are indicated above with the CBP/p300-binding site. (F) MITF DNA-binding domain-DNA co-crystal structure showing the MITF K243-DNA phosphate-backbone contact. (G) Conservation of K243 between bHLH and bHLH-LZ family members. (H) Peptide array containing indicated residues as 14-amino-acid peptides immobilized on a cellulose membrane probed with rabbit anti-acetyl-K243 antibody. (I) Western blot using anti-acetyl K243 or anti-MITF antibodies of immunoprecipitated GFP-MITF expressed alone or with co-transfected CBP or p300. (J) Western blot using anti-acetyl K243 or anti-MITF antibodies of HIS-tagged MITF purified with nickel beads. All samples were from the same blot. See also Figure S2.

The CBP and p300 lysine acetyl transferases are cofactors for a wide range of transcription factors and are proposed to facilitate transcription activation via histone acetylation. However, CBP and p300 also acetylate transcription factors (Boyes et al., 1998, Bricambert et al., 2010, Ceseña et al., 2007, Daitoku et al., 2004, Giandomenico et al., 2003, Gu and Roeder, 1997, Perrot and Rechler, 2005, Ponugoti et al., 2010). As MITF interacts with CBP and p300 (Price et al., 1998, Sato et al., 1997), we asked whether MITF is acetylated. Examination of MITF acetylation was initially frustrated by an inability to extract the protein from the nuclei of IGR37 or 501mel melanoma cells; using increasingly stringent conditions, including lysonase to digest nucleic acid or 2 M NaCl, the great majority of MITF was retained in the nuclear pellet (Figure S2A). By contrast the bHLH-LZ factor USF or the POU domain transcription factor BRN2 were readily extracted. To circumvent this problem, we co-expressed epitope-tagged MITF with CBP or p300 to generate sufficient soluble MITF for immunoprecipitation. Western blotting using anti-acetyl lysine antibody after immunoprecipitation revealed both CBP and p300 substantially increased MITF acetylation (Figure 2A).

Since the MAPK pathway is frequently activated in melanoma via mutation of BRAF or NRAS and p300 acetyl transferase activity is enhanced by MAPK-mediated phosphorylation in keratinocytes (Chen et al., 2007), we asked whether MAPK signaling activates acetylation in melanoma. Stimulation of melanoma cells with 12-O-tetradecanoylphorbol-13-acetate (TPA) transiently activated MAPK signaling detected using anti-phospho-ERK antibody, followed by a prolonged increase in histone H3 acetylation (Figure 2B). Cells were also transfected with expression vectors for Myc-epitope-tagged BRAF^V600E^, HA-tagged p300, or both, and whole-cell extracts were probed with anti-acetyl lysine antibody. Whereas p300 increased acetylation compared to untransfected cells, no increase was observed in cells transfected with BRAF^V600E^ alone, though phospho-ERK levels were increased (Figure 2C). By contrast, co-transfection of BRAF^V600E^ together with p300 dramatically increased global acetylation. In agreement, MITF acetylation driven by either p300 or CBP was blocked using the MEK inhibitor U0126 (Figure 2D). These data suggest that MITF acetylation is increased by activation of MAPK signaling, a hallmark of melanoma.

Mass spectrometry of immunoprecipitated Myc-tagged MITF revealed acetylation on five lysines (K21, K33, K43, K243, and K248) (Figures 2E, S2B, and S2C). Significantly, the MITF-DNA co-crystal structure (Pogenberg et al., 2012) revealed that K243, located at the 3′ end of the bHLH-LZ loop, makes a phosphate backbone contact but does not bind any base (Figure 2F). MITF K243 is highly conserved between species and most bHLH and bHLH-LZ transcription factors (Figure 2G) and makes a similar phosphate-backbone contact in all available crystal structures (Figure S2D). By contrast, K248 does not contact the DNA and is poorly conserved, except in the MITF family. Molecular modeling (Figure S2E) suggested that acetylation of K243 in MITF would disrupt the phosphate backbone contact. As such, acetylation of K243 should reduce the affinity of MITF for DNA without directly altering its target specificity.

To confirm acetylation of MITF K243, we generated an anti-acetyl-MITF-K243 antibody. Using an array of MITF peptides containing acetyl and non-acetyl residues corresponding to K243 or other acetylated lysines (K21, K33, and K43), control MITF residues (K205 and K206), or histone H3 peptides containing K27 and K9 (Figure 2H), we determined that the antibody was largely specific for the acetyl-K243 residue. The anti-acetyl-MITF-K243 antibody efficiently recognized immunoprecipitated MITF-GFP co-expressed with p300 or CBP (Figure 2I) or stably expressed polyhistidine-tagged MITF (purified using nickel beads after urea extraction) without ectopic p300 or CBP (Figure 2J). Note that despite extensive mass spectrometry analyses for this and other studies, we have not identified any other post-translational modification on this residue.

**Figure 3:**
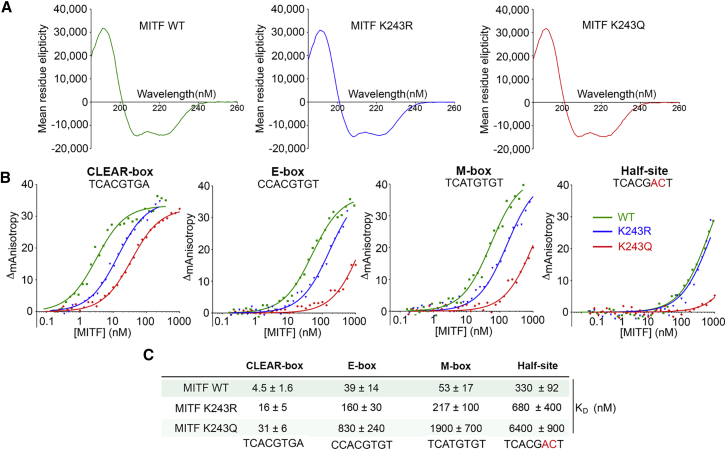
K243 Status Determines MITF DNA-Binding Affinity (A) Comparison of circular dichroism (CD) spectra of bacterially expressed and purified MITF WT and mutant DNA-binding domains. The mean residue ellipticity is plotted in dg × cm^2^ × dmol^−1^ against the wavelength (in nm). CD spectra show the mutations cause no major structural changes. (B) DNA-binding affinity of bacterially expressed and purified MITF WT and mutant DNA-binding domains determined using fluorescence anisotropy. Representative titration curves of each fluorescein-labeled oligonucleotide with MITF WT and mutants. The anisotropy values are the average of triplicate measurements from which the baseline corresponding to the anisotropy of the free fluorescent probe was subtracted. (C) The dissociation constants of MITF WT and mutants on oligonucleotides containing four different recognition sequences determined by fluorescence anisotropy.

To confirm that K243 impacts MITF DNA-binding affinity, we bacterially expressed and purified the wild-type (WT) MITF DNA-binding domain as well as K243R and K243Q mutants. Molecular modeling (Figure S2E) predicted that a K243R mutant would maintain the phosphate backbone contact, whereas the glutamine substitution, widely used as an imperfect acetylation mimetic (Mishra et al., 2016, Wang and Hayes, 2008), would break the contact and consequently impair DNA binding. Characterization of the proteins by circular dichroism indicated that both mutants had a similar structure to the WT (Figure 3A).

We next used fluorescence anisotropy to determine the *in vitro* DNA-binding affinity of all three proteins on four different binding sites: a full consensus 8-bp TCACGTGA motif termed a coordinated lysosomal expression and regulation (CLEAR) box, a CACGTG E-box lacking the key flanking 5′T and 3′A residues, a TCATGTGT M-box motif associated with the tyrosinase promoter, and a mutated (half-site) CLEAR box as a negative control. The DNA-binding data (Figure 3B) and summary of affinities (Figure 3C) revealed the WT protein bound the CLEAR box with the highest affinity and that MITF exhibited around an 11-fold reduced affinity for the differentiation-associated M-box element. Compared to WT MITF, the K243R mutant bound ∼4-fold less well on all sites. The DNA-binding affinity of the K243Q mutant was further reduced compared to the K243R mutant. K243Q bound the CLEAR box almost 7-fold less well than the WT protein and 2-fold less than the K243R mutant. Binding by the K243Q mutant was 21-fold reduced compared to the WT on an E-box and 35-fold reduced compared to the WT on an M-box, and it exhibited 5-fold and 8.7-fold reduced binding to these elements, respectively, compared to the K243R mutant. The binding affinity of all three proteins was further reduced when using the mutated CLEAR box element, but it was especially low for the K243Q mutant. In summary, the *in vitro* DNA-binding affinities of the WT and K243R and K243Q mutants confirmed that K243 contributes to the DNA-binding affinity of MITF and that the K243Q acetylation mimetic reduced DNA-binding affinity substantially more than the K243R mutation.

### K243 Plays a Critical Role in Development and Tumorigenesis

**Figure 4:**
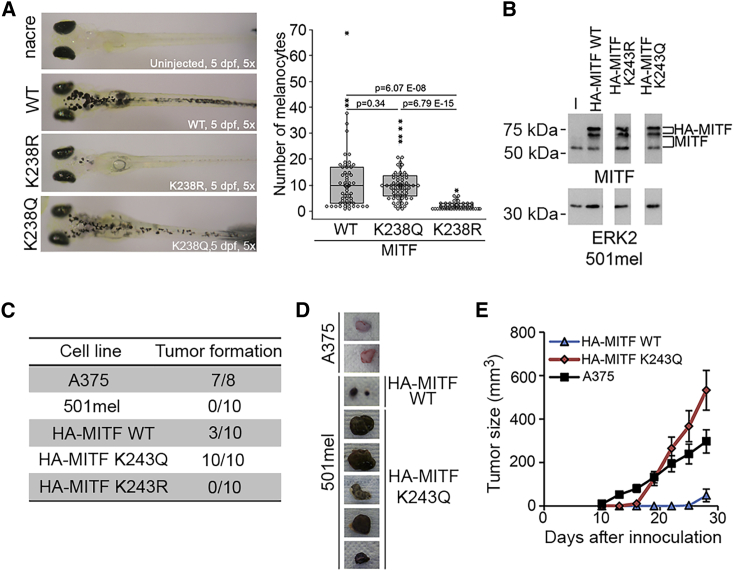
K243 Controls MITF Function *In Vivo* (A) Complementation of neural crest *MITFa-*null *nacre* zebrafish using MITF WT and K238 (equivalent to K243 in human MITF) mutants (left) and quantification of numbers of melanocytes (right). The dots in the plots represent numbers of melanocytes in each rescued embryo with at least one melanocyte. See also Figure S3. (B) Western blot of 501mel cells stably expressing HA-MITF WT and mutants (from the same gel). (C) Tumor formation after subcutaneous inoculation of indicated cell lines into athymic nude mice. (D) Example tumors. (E) Tumor size over time using indicated cell lines. Error bars indicate S.E.M.

To investigate the impact of K243 status *in vivo*, we used MITF WT or mutants to complement the absence of MITF in an *mitfa*-null *nacre* zebrafish. As expected, WT *mitfa* restored melanocyte numbers (Figures 4A and S3). However, surprisingly, the high-affinity DNA-binding K243R mutant (K238 in fish) was inactive, whereas the low-affinity K243Q mutant effectively complemented the *nacre* mutation. Thus, paradoxically, the K243Q mutant that binds DNA *in vitro* substantially less well than the K243R mutant functions much better *in vivo*.

In addition to its role in melanocyte development, MITF also plays a key pro-proliferative role in melanoma (Goding and Arnheiter, 2019, Carreira et al., 2006, Garraway et al., 2005, Giuliano et al., 2010, Hoek and Goding, 2010, Widlund et al., 2002). We therefore asked whether the status of K243 would affect melanoma growth by stably expressing HA-tagged MITF WT and the K243R and K243Q mutants in the poorly tumorigenic 501mel human melanoma cell line. Western blotting revealed that the ectopic MITF, which runs as two bands corresponding to hyper- and hypo-phosphorylated forms, was expressed ∼2- to 3-fold more than the endogenous protein (Figure 4B). In culture, we were unable to detect major differences in gene expression or proliferation rate between cells expressing ectopic WT or mutant MITF (not shown), likely owing to the expression of the endogenous MITF. However, *in vivo* endogenous MITF can be transcriptionally downregulated in xenograft tumors (Thurber et al., 2011) as well as in human melanoma (Goodall et al., 2008). We therefore asked whether the cell lines expressing WT MITF or the K243R or K243Q MITF mutants exhibited differential tumor-forming potential following subcutaneous injection into athymic nude mice. The results showed that parental 501mel cells failed to form tumors, whereas 501mel cells expressing HA-tagged WT MITF formed tumors in 3 out of 10 cases (Figures 4C–4E), consistent with MITF promoting tumorigenesis. Cells expressing the MITF K243R mutant failed to form tumors, but by contrast, K243Q-expressing cells formed large rapidly growing tumors in 10 out of 10 cases. The A375 melanoma cell line was used as a positive control. Thus, MITF K243 status is a major determinant of melanoma tumorigenicity as well as melanocyte development. Note that whereas in the zebrafish assay or cells in culture, the similar behavior of the WT and K243Q mutant suggests functional MITF may be highly acetylated, in the tumor-formation assay, WT MITF has an intermediate phenotype between the K243R and the K243Q mutants. This may indicate that WT MITF in the tumor-formation assay may be only partially acetylated, perhaps because the microenvironment after subcutaneous injection is very different from that in development or 501mel cells in culture.

### K243 Status Determines the Genome-wide Distribution of MITF

The contrasting biological output from the K243R and K243Q mutants was unlikely to arise because of differential co-factor interactions, since mass spectrometry analysis (not shown) failed to identify substantial differences in their associated proteins. We therefore hypothesized that the K243R and K243Q mutants would exhibit differential DNA binding *in vivo*. To test this, we performed an initial ChIP-seq experiment on human 501mel melanoma cells stably expressing similar levels of HA-epitope-tagged MITF WT and K243R or K243Q mutants (Figure S4A), an approach used previously to show altered targeting of the melanoma-associated MITF E318K mutant (Bertolotto et al., 2011). Some genes such as *HEXA*, which contains two MITF target sites, exhibited little difference in occupancy between WT and K243R or K243Q mutants (Figure S4A, left). By contrast, the MITF K243R mutant, but not K243Q, bound less well than WT to differentiation-associated target genes such as *MLANA* (Figure S4A, right). These data provided an indication that on some genes, the K243R mutant exhibited impaired binding compared to the lower affinity K243Q mutant.

To eliminate the possibility that the 2- to 3-fold elevated MITF WT or mutant levels expression in the cell lines used for the ChIP-seq could affect the outcome, we engineered 501mel cells to express doxycycline-inducible HA-tagged MITF WT and K243 mutants. Western blotting (Figure S4B) using different concentrations of doxycycline revealed that the levels of ectopic WT and mutant MITF could be titrated. At 0 ng doxycycline, the ectopic MITF proteins were barely detectable, and at 20 ng, the levels were similar to the endogenous protein. Immunofluorescence showed that all three proteins were largely nuclear (Figure S4C).

**Figure 5:**
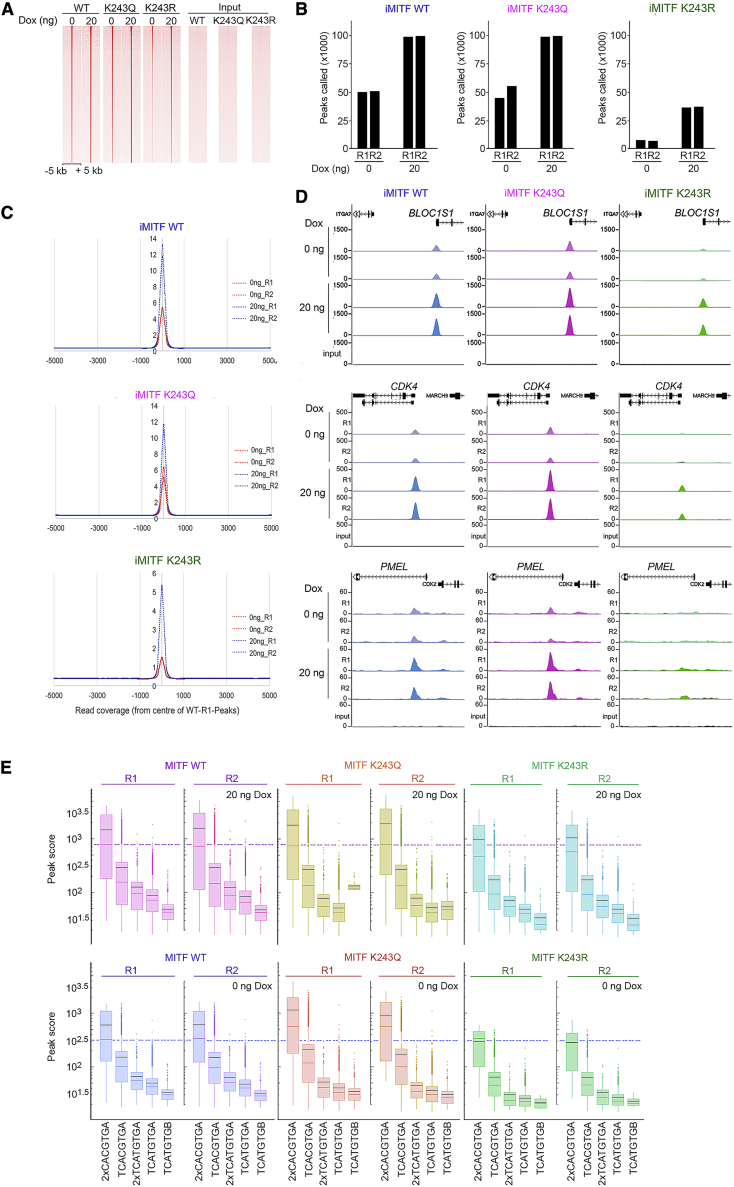
K243 Status Determines MITF Genome-wide Distribution (A) Heatmap of MITF WT and K243 mutant average tag density derived from two biological replicate ChIP-seq experiments of HA-tagged MITF expressed using 0 or 20 ng doxycycline centered on WT occupied regions (20 ng doxycycline). (B) Numbers of ChIP peaks called using HA-tagged MITF WT or mutants induced using 0 or 20 ng doxycycline. See also Table S2. (C) Read coverage of two replicates for each of the WT and K243 mutant ChIP-seq experiments expressed using 0 or 20 ng doxycycline centered around peak coordinates of the WT at 5-bp binning intervals. Numbers on the x axis indicate distance from center of the peak (in bp). (D) Genome browser screenshots of indicated loci showing HA-tagged WT and mutant MITF ChIP-seq profiles from iMITF cell lines expressing HA-tagged MITF at 0 or 20 ng doxycycline as indicated. (E) Box and whisker plots showing peak score for two replicate (R1 and R2) ChIP-seq experiments for the WT and two K243 mutants related to the indicated motifs. Expression of HA-MITF WT and mutants induced at 0 or 20 ng doxycycline. Colored line indicates median, and black line indicates mean. See also Figure S4.

Using the inducible cell lines, we repeated the ChIP-seq using the WT and mutant HA-tagged MITF proteins at both 0 and 20 ng doxycycline, with replicates exhibiting a high level of reproducibility (Figure S4D). Characterization of the ChIP efficiency and ratio of peaks called/input indicated that while the K243Q mutant behaved similarly to the WT protein, the K243R mutant bound less well (Figure S4E). Examining peak distribution also showed similar binding by the WT and K243Q mutant. The increase in expression from 0 to 20 ng doxycycline was reflected in increased binding genome-wide (Figure 5A). Again, binding by the K243R mutant at 0 ng doxycycline was severely diminished compared to the WT or K243Q mutants, but it was increased at 20 ng. The total number of peaks called using WT MITF at 0 ng was close to 50,000, which increased to ∼100,000 at 20 ng, confirming that increasing MITF expression leads to occupancy of more sites (Figure 5B). Similar results were obtained with the K243Q mutant. By contrast, the K243R mutant bound much less well, and even at 20 ng doxycycline, the number of peaks was ∼30% of the WT or K243Q mutant. The average read density centered on the peaks called revealed similar results for WT MITF and K243Q mutant, but the peak scores for the K243R mutant were reduced ∼4-fold and 3-fold at 0 and 20 ng, respectively (Figure 5C).

Using the H3K27Ac epigenetic modification as a marker of enhancers (Verfaillie et al., 2015) and 1 kb distance from the transcription start site as promoter location, we found no differences in distribution of MITF WT or K243Q mutant. The great majority of binding sites at both 0 or 20 ng doxycycline were located either in enhancers or in non-promoter locations (Figure S4F). A slightly different profile was observed using the K243R mutant, where at 0 ng doxycycline, increased relative occupancy of promoter and enhancer sites was observed, but at 20 ng, the profile was similar to that of the WT and K243Q mutant. The different distribution of K243R at 0 ng is most likely related to the greatly reduced number of peaks called (Figure 5B).

Examining individual MITF-regulated genes again revealed that WT and MITF K243Q mutant bound similarly, but the K243R mutant exhibited reduced binding, especially at those genes with the lower affinity TCATGTG M-box elements such as *PMEL* or *DCT* (Figures 5D and S4G). Comparing binding to different motifs (Figure 5E) revealed that at 0 ng doxycycline, the mean peak score was lower for K243R than WT, while that of the K243Q mutant was higher, especially for genes bearing two CLEAR-box motifs. At 20 ng doxycycline, where HA-tagged MITF was expressed at levels similar to endogenous MITF in the parental cell line (Figure S4B), binding by K243Q and WT MITF was similar at all motifs, but the K243R mutant again bound less well to all classes of binding site.

### Single-Molecule Tracking (SMT) of MITF Binding in Cells

Since the read density of the ChIP-seq experiments using MITF WT and the two mutants was normalized to the total mapped reads, the reduced peak heights associated with the K243R mutant means that there is an increase in reads distributed elsewhere within the genome that are not within peaks that pass the statistical threshold in the peak-calling algorithm. This result was unlikely to be an artifact, as it was highly reproducible across multiple ChIP-seq experiments using different concentrations of doxycycline (Figures 5 and S4D–S4G) as well as in the non-inducible stably expressing cell lines (Figure S4A).

**Figure 6:**
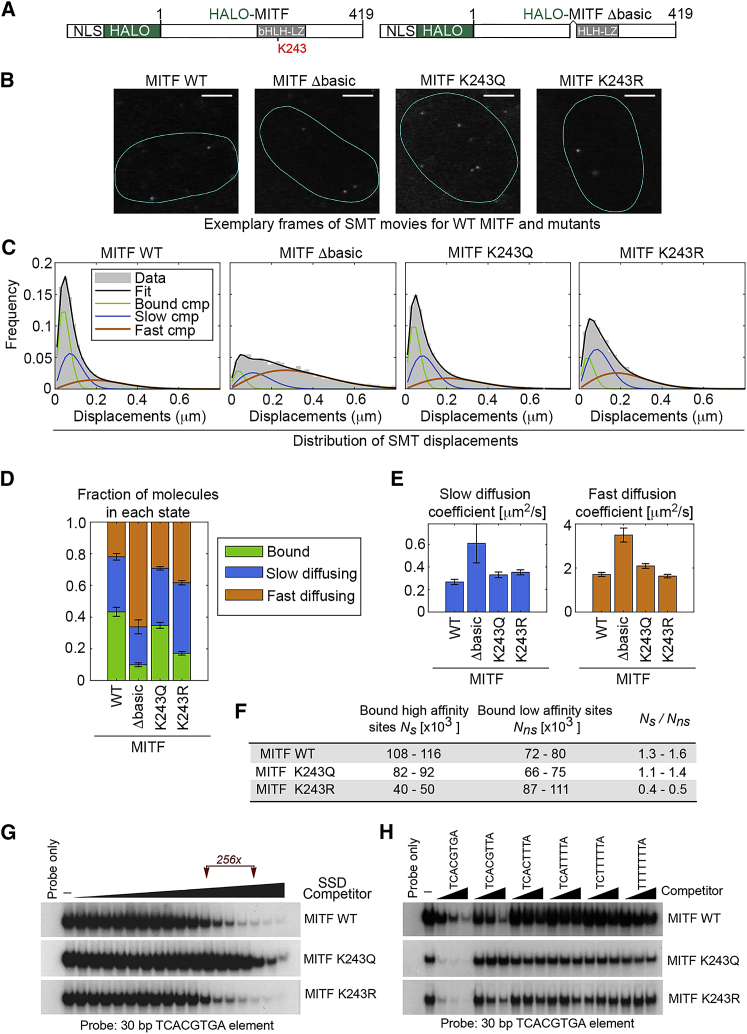
Live-Cell Single-Molecule Tracking (SMT) of HALO-Tagged MITF (A) HALO-tagged MITF expression vectors. NLS indicates the nuclear localization sequence. Δbasic lacks residues required for DNA binding. (B) Exemplary frames of SMT movies using WT and mutant HALO-tagged MITF, collected at 100 fps (see also Videos S1, S2, S3, and S4). Scale bar, 5 μm. Labeling with 100 pM Halotag JF 594 allows particle densities in the range of a few molecules per frame. (C) SMT movies were tracked to generate a distribution of single-molecule displacements between consecutive frames that was fit with a three-component model (one immobile component and two diffusing components) to provide quantitative estimates for WT MITF and mutants shown in (D) and (E). Cmp, component. (D) Quantitative estimates derived from SMT using WT and mutant HALO-tagged MITF for the fraction of molecules in each state. Error bars indicate SD. (E) Quantitative estimates of the diffusion coefficients of free molecules. For MITF WT, Δbasic, K243Q, and K243R, respectively,$\;N_{cells}=20,\;6,\;15,\;15;\;N_{displacements}=17802,\;2684,\;16422,\;12999$. Error bars indicate SD. (F) Summary derived from the SMT analysis of proportion of MITF calculated to bind high- versus low-affinity sites. (G) Electrophoretic mobility shift assay (EMSA) using bacterially expressed and purified WT and mutant MITF DNA-binding domains, a 30-bp TCACGTGA-motif-containing probe, and competition with 4-fold dilutions of SSD (10 μg to 2.3 fg). Bound DNA is shown. Probe was in excess in all reactions. (H) EMSA as in (G) with competition by indicated competitor oligonucleotides at 3, 10, and 30 ng. Bound DNA is shown. See also Figures S5 and S6 and Videos S1, S2, S3, and S4.

In an orthogonal approach to understanding the paradoxical discordance between *in vitro* binding by the K243 mutants and their binding *in vivo*, we used SMT to examine the kinetics of WT and mutant MITF binding to chromatin in live cells. In this approach (Tokunaga et al., 2008), HALO tagged-transcription factors are labeled with sub-saturating concentrations of a bright, photostable fluorescent ligand, JF594 (Grimm et al., 2015). This allows an estimation of the diffusion properties of labeled molecules, the fraction of transcription factor immobilized in the nucleus, and the duration of the binding events (Gebhardt et al., 2013, Mazza et al., 2012, Mazza et al., 2013). To this end, we generated 501mel cells stably expressing doxycycline-inducible HALO-tagged WT and K243 mutant MITF (Figure 6A). We also expressed a non-DNA-binding mutant of MITF (Δbasic) lacking the basic region required for sequence-specific DNA binding (Fock et al., 2019). Because the basic region contributes to MITF nuclear localization (Fock et al., 2019), we fused HALO-tagged WT and mutant MITF to the SV40 T-antigen nuclear localization signal (Kalderon et al., 1984). After induction of MITF expression using 20 ng doxycycline, nuclei expressing HALO-MITF were imaged (Figure 6B; Videos S1, S2, S3, and S4).

Video S1. Exemplary SMT Movie on WT HALO-Tag-MITF WT, Related to Figure 6 Frame Rate 100 Hz, Scale bar 5 μm.

Video S2. Exemplary SMT Movie on HALO-Tag-MITF ΔBasic, Related to Figure 6 Frame Rate 100 Hz, Scale bar 5 μm.

Video S3. Exemplary SMT Movie on HaloTag-MITF K243Q, Related to Figure 6 Frame Rate 100 Hz, Scale bar 5 μm.

Video S4. Exemplary SMT Movie on HaloTag-MITF K243R, Related to Figure 6 Frame Rate 100 Hz, Scale bar 5 μm.

We modeled the distribution of single-molecule displacements for WT MITF tracks collected at 100 frames per second (fps) with a three-component diffusion model (Hansen et al., 2018, Loffreda et al., 2017, Speil et al., 2011) (Figure 6C) and determined that 43% of molecules were immobilized, 34% were found in a slow diffusion state with $D_{slow}\;=\;0.34\;\text{μ}{\text{m}}^{2}/\text{s}$, and the remaining molecules were in a fast diffusion state with $D_{fast}=1.7\;\text{μ}{\text{m}}^{2}/\text{s}$ (Figure 6D). Transcription factors explore the nucleus with diffusion coefficients of this order of magnitude, which is ∼3- to 10-fold slower than expected for inert tracers of comparable sizes. Such slowdown in diffusion has been interpreted as an effect of molecular crowding in the nucleus (Izeddin et al., 2014, Mazza et al., 2012) or transient nonspecific interactions with chromatin on a timescale faster than the acquisition frame rate (Elf et al., 2007, Michelman-Ribeiro et al., 2009), a phenomenon known as effective diffusion (Sprague et al., 2004).

To interpret the measured immobile fractions and diffusion coefficients, we repeated the SMT analysis using the MITF-Δbasic mutant (Figures 6B–6D). MITF-Δbasic displayed a 4-fold drop in its immobile fraction, indicating that the WT-MITF-bound fraction largely arises owing to its interactions with DNA-binding sites. The duration of these stable MITF-binding events typically last for several seconds (Figure S5A), as reported for the majority of transcription factors studied by SMT (Chen et al., 2014, Gebhardt et al., 2013, Loffreda et al., 2017, Sugo et al., 2015, Teves et al., 2016). The slow and fast diffusion coefficients of the diffusing populations of MITF-Δbasic were ∼2-fold higher than that observed for WT MITF, supporting the hypothesis that diffusing WT molecules are slowed down by low-affinity binding events lasting a few milliseconds at most (Figure 6E).

We next performed SMT on cells expressing the HALO-MITF K243Q and K243R mutants. While the K243Q mutant displayed a minor decrease in immobile fraction (<20%) (Figures 6C and 6D), the MITF K243R mutant exhibited a more pronounced drop in its immobile fraction (2.5-fold) and a corresponding increase in the effective slow and fast diffusing fractions (Figures 6C and 6D). However, surprisingly, SMT did not reveal any substantial difference in the residence time of MITF WT and K243 mutants (Figures S5A–S5D). Thus, compared to the WT and K243Q mutant, the K243R mutant exhibits a reduced immobile fraction but a similar residence time and diffusion coefficient (Figure 6E). The SMT data therefore support the ChIP observations; K243Q behaves dynamically very similarly to WT MITF (although with a marginally reduced immobile fraction), whereas the K243R mutant has its binding equilibrium shifted toward an unbound or transiently bound (effective diffusion) state. In other words, by binding transiently but frequently to low-affinity sites genome-wide, the K243R mutant is less able to bind higher affinity sites associated with gene regulation.

If we assume that the measured immobile fraction by SMT reflects specific long-lived binding and that the slowdown in diffusion (compared to MITF-Δbasic) is due to nonspecific transient binding, we can use the estimates from the SMT data to calculate the number of high-affinity and low-affinity binding sites bound at any time. By this calculation, long-lived binding events occur 1.5 times more frequently than transient binding events for WT MITF (Figure 6F). This ratio is slightly reduced for MITF-K243Q (1.1–1.4 times), while it is 3-fold lower for MITF-K243R (0.4–0.5 times). These data are in very good agreement with the ChIP-seq performed using WT and mutant MITF.

To confirm these data, we also performed an SMT-based competition assay (Figure S6A). Using a cell line stably expressing WT HALO-MITF, we introduced expression vectors for doxycycline-inducible GFP-tagged WT and K243R and K243Q mutants and determined the bound and slow- and fast-diffusing fractions. The results (Figures S6B and S6C) indicate that the bound fraction of the stably expressed HALO-MITF WT is substantially reduced when the K243Q mutant is expressed, but not when the K243R mutant is expressed. The WT exhibits an intermediate effect, consistent with it being partially acetylated.

As an additional control, we also determined the bound and slow- and fast-diffusing factors for a stably expressed MITF mutant bearing a quadruple K > R substitution in the four additional acetylation sites identified here (K21, K33, K43, and K248) (Figure S6D). The 4KR mutant exhibited a similar distribution of displacements (Figure S6E), bound (Figure S6F) and fast diffusing coefficient (Figure S6G) as the WT MITF. This indicates that these additional acetylated residues are unlikely to contribute to MITF DNA-binding activity. This result was confirmed using ChIP-PCR to compare occupancy of WT MITF to that of the 4KR mutant on a set of MITF-binding sites. No significant differences in occupancy were apparent (Figure S6H).

### K243 Status Dictates Titration by Genomic DNA

To characterize the equilibrium between MITF specific and nonspecific binding *in vitro*, we performed a DNA-binding electrophoretic mobility shift assay using a radiolabeled probe containing a high-affinity TCACGTGA MITF recognition site together with bacterially expressed and purified MITF WT and K243 mutants. Binding to the probe was challenged with increasing amounts of salmon sperm genomic DNA (SSD) as competitor to reflect the presence of genomic DNA present within nuclei. The results showed that bacterially expressed WT MITF (non-acetylated) or K243R mutant were similarly competed by the SSD from binding the consensus TCACGTGA probe (Figure 6G). By contrast, ∼256-fold more SSD was required to similarly compete the K243Q mutant. All three proteins were competed well by a cold TCACGTGA consensus competitor, and mutations in more than one base of this motif prevented binding (Figure 6H). However, the K243R and WT, but not the K243Q, proteins were also competed by a low-affinity TCACGTTA motif. These result can be explained by higher affinity binding by the K243R mutant conferred by the additional phosphate-backbone contact enabling it to bind the genome-wide excess of weaker binding sites that diverge from the consensus. Thus the *in vitro* DNA-binding assays are in agreement with both the SMT and ChIP-seq analyses, and collectively, the data confirm the paradoxical result that a transcription factor with reduced DNA-binding affinity *in vitro* (MITF K243Q) can function better *in vivo* than a higher affinity binding protein (K243R).

Finally, for at least two transcription factors, p48 (PTF1A; involved in pancreatic acinar cell differentiation; Rodolosse et al., 2009) and the neuronal differentiation 1 (NEUROD;BETA-2) bHLH factor (implicated in pancreatic islet cell development and function; Qiu et al., 2004), the equivalent lysine is acetylated and implicated in driving gene expression. We therefore established a stable cell line expressing similar levels of HALO-tagged NEUROD WT or K138R or K138Q mutants (K243 equivalent) and performed SMT analysis. The results revealed that WT NEUROD exhibited a distribution of displacements that lay between that of the K138R and K138Q mutants (Figure S6I). As with the MITF K243R mutant, the bound fraction of the K138R mutant was reduced compared to the WT and K138Q mutant, with the latter also exhibiting a reduced fast-diffusing fraction (Figure S6J) and reduced fast diffusion coefficient (Figure S6K). Collectively, these data suggest that the modification status of K138 in NEUROD plays a similar role in dictating the effective available fraction of the transcription factor as K243 in MITF.

## Discussion

Models of transcription regulation suggest that transcription factors engage in extensive nonfunctional interactions within chromatin-accessible regions (Fisher et al., 2012) and that binding to regulatory elements is intrinsically stochastic and highly dynamic (Liu and Tjian, 2018, McNally et al., 2000). Moreover, the probability of transcription initiation occurring is largely determined by the affinity of the binding site for the transcription factor (Segal and Widom, 2009). It is therefore widely assumed that decreasing transcription factor DNA-binding affinity reduces transcription initiation by diminishing occupancy of the sequence-specific regulatory elements. However, our results, obtained using MITF as a model, highlight an unexpected paradox. The acetylation mimetic K243Q mutant exhibits reduced DNA-binding affinity *in vitro* but supports melanocyte development and tumor formation better *in vivo* than the non-acetylatable higher affinity K243R mutant. The WT MITF protein has a similar affinity for DNA as the K243R mutant *in vitro*, but, consistent with WT MITF being acetylated in cells, it instead supports melanocyte development like the lower affinity K243Q mutant. The biological activity of the WT and mutants is reflected in their relative ChIP efficiency, with WT and low-affinity K243Q mutant binding better in cells than the high-affinity K243R mutant. Moreover, as expected, increasing levels of MITF WT and mutants led to increased occupancy of binding sites genome-wide, reflected in both increased peak height and more sites bound, though at physiological or sub-physiological levels, the difference between the K243R mutant and the WT/K243Q mutant was maintained. Explaining the paradox that reduced DNA binding affinity *in vitro* translates to better DNA binding and function *in vivo* provides a novel and unanticipated insight into transcription factor dynamics.

Counterintuitively, we propose that the simplest and most likely explanation for the differential recognition of binding sites *in vivo* by the WT/K243Q and K243R proteins is their differential DNA-binding affinity; a genome-wide excess of very low-affinity binding sites may be bound by a high-affinity transcription factor such as the K243R mutant but may not be recognized effectively by either the K243Q mutant or an acetylated WT protein. In this model, an excess of low-affinity sites will reduce the effective concentration of MITF that is free to recognize the productive binding sites that support melanocyte development and tumor formation. This explanation was supported by our observation that *in vitro*, in the absence of any co-factors, SSD titrated the high-affinity unacetylated WT protein and the K243R mutant better than the low-affinity K243Q mutant, leaving the K243Q mutant better able to bind a consensus binding site. Such a model to control transcription factor availability was originally hypothesized for the bacterial Lac repressor (von Hippel et al., 1974), and more recently, it was shown that in *E. coli*, competing LacR-binding sites on plasmids whose copy numbers vary during the cell cycle can have a substantial impact on the dosage response to that transcription factor (Brewster et al., 2014).

While a large excess of degenerate, low-affinity motifs presents a thermodynamic challenge to a given transcription factor locating and remaining at its target regulatory elements, it also offers an opportunity for regulation. However, for a regulated genomic titration model to be valid, the number of molecules of a transcription factor able to bind DNA should not be in large excess over the number of potential binding sites. In mammalian cells, transcription factor copy numbers vary widely (Biggin, 2011), but the fact that increasing transcription factor expression levels cause changes in gene expression and that limiting numbers of transcription factors can be redirected to alternative sites by binding partners (Hosokawa et al., 2018) implies that they are not effectively in excess over their accessible DNA-binding sites. By contrast, early observations made using fluorescence recovery after photobleaching (FRAP) suggested that the great majority of transcription factor molecules in a cell are fast diffusing and are therefore potentially in excess over their binding sites (McNally et al., 2000, Sprague et al., 2004, Stenoien et al., 2001). However, as a population technique, FRAP may fail to dissect the behavior of multiple coexisting populations (e.g., stably bound, transiently bound to nonspecific sites, or freely diffusing). Instead, we used SMT to address the dynamic behavior of MITF WT and the K243 mutants. The results were consistent with the ChIP-seq data as well as the *in vitro* DNA binding in the presence of salmon sperm DNA. SMT movies with a frame rate of 100 fps revealed that 45% of MITF WT was immobile, a fraction higher than SOX2 (23%) or steroid receptor (20%–40%), while slower acquisitions determined an average residence time of 6 s, similar to that of other transcription factors (Chen et al., 2014, Gebhardt et al., 2013, Loffreda et al., 2017, Sugo et al., 2015, Teves et al., 2016, Morisaki et al., 2014, van Royen et al., 2011). Surprisingly, although the MITF non-DNA-binding mutant exhibited a large decrease in the immobile fraction, it also displayed an increase in the diffusion coefficients of the diffusing populations. This indicates that ∼30% of MITF WT diffusion is interdispersed with very transient (faster than the acquisition rate) binding events to low-affinity sites that reflect the proposed genomic reservoir, resulting in slower effective diffusion coefficients. The other two MITF populations determined from the biexponential fitting of the distribution of residence times, accounting for 45% of the molecules, are representative of more stable (higher affinity) binding events. Importantly, the high-affinity K243R mutant exhibited a significantly reduced immobile fraction and an increase in both the fast- and slow-diffusing fractions compared to the WT or K243Q mutant. This reflects, in this orthogonal approach, the reduced DNA binding by MITF K243R observed using ChIP-seq, and the data are consistent with a greater proportion of the high-affinity K243R mutant binding transiently to very low-affinity sequences genome-wide, thereby reducing the pool of molecules able to target sites bound better by the WT or K243Q mutant. Since the bound fraction determines transcription burst frequency (Stavreva et al., 2019), our results are consistent with the K243Q or acetylated WT MITF, but not the K243R mutant, supporting melanocyte development and promoting tumor growth. Notably, in development, as shown by the ChIP-seq and SMT assays, the MITF WT protein behaves much more like the K243Q mutant than the K243R mutant, suggesting that under the conditions used, a significant proportion of functional MITF is acetylated. However, it is important to note that additional post-translational modifications, including acetylation on other residues not identified here, by p300/CBP or possibly other acetyl transferases, or non-acetylation modifications such as sumoylation, phosphorylation, or methylation might also affect MITF target specificity.

The increased binding observed after induction of MITF expression using doxycycline is consistent with the rheostat model for MITF function (Carreira et al., 2006) that suggests that increasing MITF activity changes its corresponding gene expression program. However, MITF “activity” is defined as a combination of the amount of protein in a cell and post-translational modifications that affect function. Since we found no evidence that mutation of K243 affects MITF protein levels, K243 acetylation status must control the effective concentration of MITF by regulating its availability within cells. The K243-Ac/K243Q is unable to bind the genome-wide excess of very low-affinity sites and consequently has a higher effective concentration; by contrast, unmodified K243 or the K243R mutant has a lower effective concentration and less availability to bind key regulatory elements, as it instead binds an excess of low-affinity sites genome-wide. Since we show that CATGTG M-box motifs associated with differentiation genes exhibit lower affinity for MITF than CACGTG elements, we anticipate that transcriptionally productive recognition of lower affinity sites may require higher effective levels of MITF that may be achieved by a combination of K243 acetylation as well as increased protein levels, for example in response to MC1R signaling following UV irradiation (Cui et al., 2007, Price et al., 1998, Malcov-Brog et al., 2018).

For the bHLH or bHLH-LZ family, acetylation by p300/CBP, or potentially other lysine acetyl-transferases, such as p300/CBP-associated factor (PCAF) (Rodolosse et al., 2009) or GCN5 (Wang et al., 2020), may regulate their DNA-binding affinity. In this respect, regulation of transcription factor acetylation may be generally important. Significantly, lysine 243 in MITF is highly conserved in most bHLH and bHLH-LZ transcription factors and makes a similar phosphate backbone contact in available crystal structures. Importantly, acetylation of the bHLH factor NEUROD at K138 (the K243-equivalent residue) enhances its capacity to regulate transcription (Qiu et al., 2004), and a K138Q mutation increases the bound fraction measured by SMT. We therefore view it as likely that acetylation-regulated genomic redistribution achieved by moderately reducing a transcription factor’s affinity for DNA may be a widespread mechanism for determining transcription factor availability. Moreover, since p300 activity can be regulated by MAPK signaling (Chen et al., 2007), the greatly enhanced tumor-forming capacity observed with the MITF K243Q mutant compared to K243R further suggests that transcription factor acetylation driven by activated oncogenes like BRAF will fuel proliferation in cancer in part by releasing transcription factors from their genomic reservoir.

In summary, by regulating a transcription factor’s affinity for DNA, the balance between the pool of molecules binding the genomic reservoir and those recognizing productive binding sites may be controlled. Our results suggest that competition by an excess of low-affinity sites means paradoxically that moderately reducing DNA-binding affinity may be crucial for raising transcription factor availability above the threshold required for productive gene regulation underpinning development and tumorigenesis.

## STAR★Methods

### Key Resources Table

**Table:** 

REAGENT or RESOURCE	SOURCE	IDENTIFIER
**Antibodies**		

HA	Roche	Cat# 11666606001; RRID: AB_514506
HA	Sigma-Aldrich	Cat# H3663; RRID: AB_262051
HA	Cell Signaling Technology	Cat# 2367; RRID: AB_2314619
FLAG	Sigma-Aldrich	Cat# F1804; RRID: AB_262044
Acetylated-Lysine	Cell Signaling Technology	Cat# 9441; RRID: AB_331805
MITF	Millipore	Cat# MAB3747; RRID: AB_570596
ERK 2	Santa Cruz Biotechnology	Cat# sc-153; RRID: AB_2141293
ERK 2	Santa Cruz Biotechnology	Cat# sc-1647; RRID: AB_627547
Thr202/Tyr204, ppERK	Cell Signaling Technology	Cat# 4377; RRID: AB_331775
Catenin, beta	BD Biosciences	Cat# 610154; RRID: AB_397555
USF2	Santa Cruz Biotechnology	Cat# sc-862; RRID: AB_632580
Lamin B	Santa Cruz Biotechnology	Cat# sc-6216; RRID: AB_648156
GAPDH	Santa Cruz Biotechnology	Cat# sc-32233; RRID: AB_627679
Actin	Santa Cruz Biotechnology	Cat# sc-47778;RRID:AB_2714189
Histone H3	Abcam	Cat# ab1791; RRID: AB_302613
H3K27Ac	Cell Signaling Technology	Cat# 8173BF; RRID: AB_2616015
MITF-K243 specific antibody	This paper	N/A

**Bacterial and Virus Strains**		

Subcloning Efficiency DH5α bacteria	Invitrogen	Cat#18265-017
BL21(DE3)	Agilent Technologies	Cat#200131
Biological Samples	N/A	N/A

**Chemicals, Peptides, and Recombinant Proteins**		

Doxycycline	Sigma	Cat# D9891
FuGENE 6	Promega	Cat# E2692
Geneticin	GIBCO	Cat# 10131027
Puromycin	GIBCO	Cat# A1113802
MITF-recombinant proteins	In house	This paper
M344	Stratech Scientific	Cat# S2779-SEL
cOmplete ULTRA Protease Inhibitor Cocktail	Sigma	Cat# 5892988001
Urea	Sigma	Cat# 51457-100ML
Guanidine hydrochloride	Sigma	Cat# G4505-100G
Imidazole	Sigma	Cat# 792527-100G
Ni-NTA His⋅Bind® Superflow Resin	Merck	Cat# 70691
cOmplete ULTRA Tablets, Mini, EDTA-free	Sigma	Cat# 06538304001

**Critical Commercial Assays**		

QIAquick PCR Purification Kit	QIAGEN	Cat# 28106
QuikChange Lightning Site-Directed Mutagenesis Kit	Agilent Technologies	Cat# 210519
PureLink Genomic DNA Mini Kit	Invitrogen	Cat# K182002
QIAprep Spin Miniprep Kit	QIAGEN	Cat# 27106
Qubit dsDNA HS Assay Kit	Invitrogen	Cat# Q32851
Agilent High Sensitivity DNA Kit	Agilent Technologies	Cat# 5067-4626

**Deposited Data**		

ChIP-seq 501mel Human melanoma cell line ectopically expressing inducible HA-Mitf	GSE137522	This paper
ChIP-seq data of 501mel cells constitutively expressing HA-Mitf	GSE77437	This paper

**Experimental Models: Cell Lines**		

501mel Human melanoma cell line (female)	Obtained from Ruth Halaban, Yale	(Zakut et al., 1993) RRID:CVCL_4633
A375M Human melanoma cell line (male)	ATCC	(Fogh et al., 1977, Giard et al., 1973) RRID:CVCL_B222
Phoenix-Ampho Human fetal cell line (female)	ATCC	RRID:CVCL_H716
501mel Human melanoma cell line ectopically expressing inducible 3xHA-Mitf-WT	In house	This paper
501mel Human melanoma cell line ectopically expressing inducible 3xHA-Mitf-K243Q	In house	This paper
501mel Human melanoma cell line ectopically expressing inducible 3xHA-Mitf-K243R	In house	This paper
501mel Human melanoma cell line ectopically expressing inducible NLS-HTN-Mitf-WT	In house	This paper
501mel Human melanoma cell line ectopically expressing inducible NLS-HTN-Mitf-K243Q	In house	This paper
501mel Human melanoma cell line ectopically expressing inducible NLS-HTN-Mitf-K243R	In house	This paper
501mel Human melanoma cell line ectopically expressing inducible NLS-HTN-Mitf-basic	In house	This paper
501mel Human melanoma cell line constitutively expressing ectopic 3xHA-Mitf-WT	In house	This paper
501mel Human melanoma cell line constitutively expressing ectopic 3xHA-Mitf-K243Q	In house	This paper
501mel Human melanoma cell line constitutively expressing ectopic 3xHA-Mitf-K243R	In house	This paper
501mel Human melanoma cell line ectopically expressing inducible MITF-10HIS	In house	This paper
501mel Human melanoma cell line constitutively expressing ectopic HTN-3xHA-Mitf	In house	This paper
501mel Human melanoma cell line constitutively expressing ectopic HTN-3xHA-Mitf and inducible EmGFP-MITF-WT	In house	This paper
501mel Human melanoma cell line constitutively expressing ectopic HTN-3xHA-Mitf and inducible EmGFP-MITF-K243Q	In house	This paper
501mel Human melanoma cell line constitutively expressing ectopic HTN-3xHA-Mitf and inducible EmGFP-MITF-K243R	In house	This paper
501mel Human melanoma cell line constitutively expressing ectopic HTN-NEUROD1-WT	In house	This paper
501mel Human melanoma cell line constitutively expressing ectopic HTN-NEUROD1-K139Q	In house	This paper
501mel Human melanoma cell line constitutively expressing ectopic HTN-NEUROD1-K139R	In house	This paper

**Experimental Models: Organisms/Strains**		

Athymic nude mice, 7‐9 weeks old	Charles River	Crl:NU(NCr)-Foxn1^nu^
Zebrafish nacre line	Bred in house	(Lister et al., 1999)

**Oligonucleotides**		

SDM-primer K243Q 5′-CAGACATGC GGTGGAACCAGGGAACCATTCTCAAG	Eurofins	This paper
SDM-primer K243R 5′-ATCCAGACATGCG GTGGAACAGGGGAACCATTC	Eurofins	This paper
SDM -primer K21R5′- CTTGCTGTATGTGGT ACCTGGTGGGGTTTTCCAGG-3′	IDT	This paper
SDM -primer K33R5′- TAGAAAGGTAC TGCCTTACCTGGTGCCTCTGAGC-3′	IDT	This paper
SDM -primer K43R5′- CTTGGCTGGCATG TCTATTTGCTAAAGTGGTAGAAAGGTACT-3′	IDT	This paper
SDM -primer K248R5′- CCACAGAGGCCCT GAGAATGGTTCCCTTGTTCC-3′	IDT	This paper
NLS insertion-primer 5′-CGCCTAGCGGCCG CATGGCTCCGAAGAAGAAGCGGAAGGTAG CTGATACCACCATG	IDT	This paper
basic-primer 5′-GAGCATTGGCTAAAGA GAGGTTTAACATAAACGACCGCAT	IDT	This paper
SDM-primer-NEUROD1-K138Q5′- GACGCA GAAGCTGTCCCAGATCGAGACTCTGCGCT	Eurofins	This paper
SDM-primer-NEUROD1-K138R5′- CGCAG AAGCTGTCCAGAATCGAGACTCTGCG	Eurofins	This paper
Sub-cloning of pHTN-Mitf into pPB-hCMV^∗^1-cHA-pA 5′-AAAGCCACCGCGGCCGCATGGCAGAAATCGG TATTTCTAGAGCGGCCGCCTACACACCATGCTCC GTTTCTTCTGCGCTCAT	IDT	This paper
Insertion of EmGFP into pPB5′- AGACTCA CAATTG ATGGTGAGCAAGGGCGAGGAGC-3′5’ AGACTCA GAATTC TGGGCTCGAGCCCTTGTACAGCTC-3′	IDT	This paper
Insertion of HIS-tag into pPB5′- ATCGACTACAAGG ATGACGATGACAAGCATCATCACCATCACCATCA CCATCACCACTAGTGA-3′5’- GGGTAGGCCATGG CACTAGTTCATCACTAGTGGTGATGGTGATGGTG ATGGTGATGATG-3′	IDT	This paper
Genotyping primer 5′-CACAGAGTCTGAAGCAA GAGC5′-CCGGTGGATGGGATAAGGGAAAGTC	IDT	This paper
Fluorescence anisotropy probes (backbone) 5′- GAGATCACGTGATGAC-3′-Fluorescein	METABION	This paper
EMSA probe 5′-CTAGACTTGTGGAGA TCACGTGATGACTTCCTGATTCCTCompetitor probes have same backbone sequence	Eurofins	This paper
BLOC1S1_ChIP-qPCRF 5′-GATCTTCA CCCAAGGTCTCAAR 5′-AAGCTGGAACGCTCACC	IDT	This paper
CDK4_ChIP-qPCRF 5′-TGCTTCGACTGGGAGGAR 5′-CAAGCGGTCACGTGTGATA	IDT	This paper
GAPDH_ChIP-qPCR non MITF target –ve controlF 5′-GAAGGGCTTCGTATGACTGGR 5′-CTTAAG GCATGGCTGCAACT	IDT	This paper
GRIN2A_ChIP-qPCRF 5′-CTGGCCCAATTC TTCATCTCTR 5′-GTCACGTGATCAAACTCAAAGTC	IDT	This paper
GTF2H1_ChIP-qPCRF 5′-CTACTAACGGCA CTTCCTCATCR 5′-AGGCCGTAGAGAGCGTAAT	IDT	This paper
KAT5_ChIP-qPCRF 5′-GAGACGCCCGAGGACTTR 5′-CGAAGCTGGTCACGTGTATG	IDT	This paper
MTHFR_ChIP-qPCRF 5′-CCTGGTCTCA GTCCCAGAR 5′-CTTCCTCCTTTACTGCCACTC	IDT	This paper
SIRT1_ChIP-qPCRF 5′-CTCGCCACAAA GAGGAAGGR 5′-CCACAACACTACGGGTCAC	IDT	This paper

**Recombinant DNA**		

Piggybac expression system	(Magnúsdóttir et al., 2013)	(Magnúsdóttir et al., 2013)
pPB-3xHA-Mitf-WT	In house	This paper
pPB-3xHA-Mitf-K243Q	In house	This paper
pPB-3xHA-Mitf-K243R	In house	This paper
pPB-MITF-10xHIS	In house	This paper
pCMV5-3xHA-Mitf-WT	In house	This paper
pCMV5-3xHA-Mitf-K243Q	In house	This paper
pCMV5-3xHA-Mitf-K243R	In house	This paper
pPB-NLS-HTN-Mitf-WT	In house	This paper
pPB-NLS-HTN-Mitf-K243Q	In house	This paper
pPB-NLS-HTN-Mitf-K243R	In house	This paper
pPB-NLS-HTN-Mitf-Δbasic	In house	This paper
pPB-NLS-HTN-Mitf-K21/33/43/248R	In house	This paper
pPB-3xHA-Mitf-K21/33/43/248R	In house	This paper
pHTN-3xHA-Mitf	In house	This paper
pPB-EmGFP-3xHA-MITF-WT	In house	This paper
pPB-EmGFP-3xHA-MITF-K243Q	In house	This paper
pPB-EmGFP-3xHA-MITF-K243R	In house	This paper
pEZ-HTN-NEUROD1	GeneCopoeia	#EX-M0522-M49
pEZ-HTN-NEUROD1-K138Q	In house	This paper
pEZ-HTN-NEUROD1-K138R	In house	This paper

**Software and Algorithms**		

STAR v 2.5.1b, Genome GRCh38 v23	(Dobin et al., 2013)	N/A
Homer v4.9.1, Genome hg38 v5.10	(Heinz et al., 2010)	N/A
ggplot2-v3.0.0 – under R-v3.5.1		https://cran.r-project.org/web/packages/ggplot2/index.html
TreeView	(Saldanha, 2004)	N/A
Bowtie 1.1.2	(Langmead and Salzberg, 2012)	N/A
PicardTools version 1.96,		http://picard.sourceforge.net
seqMINER	(Ye et al., 2011)	N/A
deepTools	(Ramírez et al., 2014)	N/A
MACS2 v2.1.0	(Zhang et al., 2008)	N/A
CisGenome	(Ji et al., 2008)	N/A
MEME	(Machanick and Bailey, 2011)	N/A
BedTools	(Quinlan and Hall, 2010)	N/A
BWAv0.7.8	N/A	https://academic.oup.com/bioinformatics/article/25/14/1754/225615
Bioconductor	N/A	https://www.bioconductor.org

### Resource Availability

#### Lead Contact

Further information and requests for resources and reagents should be directed to and will be fulfilled by the Lead Contact, Colin R Goding (colin.goding@ludwig.ox.ac.uk).

#### Materials Availability Statement

Plasmids and cell lines generated in this study are available upon request. The K243Ac-specific antibody is of limited-availability, although we can provide information on supplier which may be used to procure additional material.

#### Data and Code Availability

The accession number for the ChIP-Seq datasets using constitutive or inducible HA-MITF reported in this paper are Gene Expression Omnibus: GSE77437, GSE137522. All bioinformatics analyses were carried out using publically available packages as described in METHOD DETAILS section. Original data have been depositied to Mendeley Data: https://doi.org/10.17632/2ccckzsk26.1. Exemplary SMT movies are provided in Videos S1, S2, S3, and S4. Additional movie files are available on request.

### Experimental Model and Subject Details

#### *In vivo* animal studies

##### Zebrafish

###### Ethical statement

All zebrafish experiments are performed in accordance with the *Animals (Scientific Procedures) Act* 1986, and approved by the University of Edinburgh Animal Welfare and Ethical Review Body.

##### Experimental procedures

Plasmid DNA (62.5 ng/μl) comprising the zebrafish *mitfa* promoter driving the fish MITF cDNA was mixed with Tol2 mRNA (70 ng/μl). 2 nL of the mixture was injected into 1-cell stage *mitf* null *nacre* embryos. Injected embryos were grown at 28°C for 5 days. On day 5, embryos were briefly exposed to white light to contract melanocytes and were then imaged before being fixed in 4% PFA. The total number of surface melanocytes in the head, trunk and yolksac was counted.

##### Experimental animals

Zebrafish AB/TPL lines were bred, raised and maintained as described (Westerfield, 2000).

##### Mouse

###### Ethical statement

Experiments were approved by the animal use ethical committee of Oxford University and fully complied with UK Home Office guidelines.

##### Experimental procedures

1 × 10^6^ cells in 100 μl PBS were subcutaneously injected into the flanks of 7-9 week old female athymic nude mice at day 1. Tumor size was measured every three days with Vernier callipers. Tumor volumes were calculated using the following formula: (L ^∗^ W ^∗^ W)/2, in which L represents the large diameter of the tumor, and W represents the small diameter. Animals were sacrificed and the tumors isolated when a tumor reached approximately 1 cm diameter, or before.

##### Experimental animals

Healthy female athymic nude mice (Crl:NU(NCr)-Foxn1^nu^) aged 7‐9 weeks were purchased from Charles River.

#### Experimental Models

##### Cell Lines

###### Human cell line information

501mel Human melanoma cell line (female, RRID:CVCL_4633)
A375M Human melanoma cell line (male, RRID:CVCL_B222)
Phoenix-Ampho Human fetal cell line (female, RRID:CVCL_H716)

All melanoma cell lines were authenticated by STR analysis using Eurofins Genomic service. All parental and derivative cell lines were verified mycoplasma free using Ludwig Cancer Research monthly mycoplasma check service.

##### Culture/Growth condition

Melanoma cell lines and their stable transfectants were grown in RPMI-1640 (GIBCO BRL, Invitrogen), supplemented with 10% fetal bovine serum (FBS, Biosera). Phoenix-ampho cells were grown in DMEM (Lonza), supplemented with 10% FBS. Cells were maintained in humidified incubator at 37°C with 10% CO_2_.

#### Bacterial Strains

*Escherichia coli* BL21(DE3) cells, Genotype: B F^–^ *dcm ompT hsdS*(r_B_^–^ m_B_^–^) *gal* λ(DE3) (Agilent Technologies #200131).

*Escherichia coli* DH5α bacteria Genotype: F^-^ Φ80*lac*ZΔM15 Δ(*lac*ZYA-*arg*F) U169 *rec*A1 *end*A1 *hsd*R17(r_k_^-^, m_k_^+^) *pho*A *sup*E44 *thi*-1 *gyr*A96 *rel*A1 λ^-^ (Invitrogen #18265017)

### Method Details

#### Plasmids

pCS2-6x Myc-MITF has been described previously (Schepsky et al., 2006). pCMV14-3 × FLAG-MITF was made by insertion of the MITF mouse cDNA from the ATG start codon, but lacking a stop codon into the vector polylinker and expresses MITF with a C-terminal triple flag epitope. pcDNA3-3 × HA-MITF was constructed by inserting the MITF cDNA from the ATG start codon to the stop codon, and pETM-11-MITFΔN180ΔC296 has been described (Pogenberg et al., 2012). The doxycycline-inducible 3xHA-Mitf plasmid was made through PCR-mediated sub-cloning of pCDNA5-3xHA-Mitf into pPB-hCMV^∗^1-cHA-pA (Magnúsdóttir et al., 2013) using EcoRI-HF (NEB; Cat#R3101L). pHTN-Mitf was sub-cloned from pCDNA5-3xHA-Mitf using EcoRI-HF. The doxycycline-inducible Halo-tag-Mitf plasmids were generated through sub-cloning of pHTN-Mitf into pPB-hCMV^∗^1-cHA-pA using NotI-HF (NEB; Cat#R3189S). The NLS derived from SV40 and 10xHIS-tag were introduced into pPB-HTN-Mitf and pPB-MITF, respectively through PCR mediated insertion and point mutations were introduced using QuikChange Lightning Site-Directed Mutagenesis Kit (Agilent; Cat# 210519). The basic domain of Mitf was removed through PCR-mediated deletion to generate the pNLS-HTN-Mitf-Δbasic construct. pPB-EmGFP-3xHA-MITF was generated through PCR mediated subcloning of EmGFP from pcDNA6.2-N-EmGFP-GW into pPB. pEF-Myc-BRAF^V600E^ was a gift from Richard Marais, pcDNA3-3 × HA-p300 was a gift from Bernhard Luescher. All plasmids were sequenced prior to use.

#### Stable cell line generation

501mel cells stably expressing HA-epitope tagged WT MITF have been described (Strub et al., 2011). 501mel melanoma cell lines ectopically expressing inducible Mitf were generated by co-transfecting pPB-3xHA-Mitf with pPB-rttA and pPB-transposase in an 8:1:1 ratio using Fugene 6 (Promega; Cat# E2692) at a 3:1 FuGENE 6:DNA ratio. Cells were initially selected for pPB-rttA using 1mg/ml Geneticin (GIBCO; Cat# 10131027) then for pPB-3xHA-Mitf with 5 μg/ml Puromycin (GIBCO; Cat# A1113802). The resulting cell lines were genotype for integration of the corrected mutant through sequencing of amplicon PCR-amplified from genomic DNA extracted using PureLink Genomic DNA Mini Kit (Invitrogen; Cat# K182002). Clonal populations were selected, dissociated and expanded before being analyzed for appropriate HA-MITF expression. 501mel cells stably expressing Halo-tag Mitf and NEUROD1 were generated through transient transfection of plasmids using Fugene 6 at a 3:1 FuGENE 6:DNA ratio for 3 rounds as the cells were successively passage from 6 cm to 10 cm dish and to T75 flask before selection in 1mg/ml Geneticin till stably ectopically expressing cell lines were obtained.

#### Western blotting

Hot LDS Sample Buffer (Invitrogen; Cat# NP0008) supplemented with β-mecaptoethanol (Sigma; Cat# M6250) was used to lyse cells before being subjected to SDS-PAGE using either a 19:1 ratio of acrylamide:bis-acrylamide, or a 200:1 ratio, as in Figure 2A, to allow better resolution of phosphorylated proteins. Note that the 200:1 ratio leads to proteins exhibiting an apparent increase in molecular weight if they are modified. Proteins transferred to Immobilon-FL polyvinylidene difluoride membranes (Millipore; Cat# IPFL00010) that were blocked with 5% non-fat milk, in TBS containing 0.1% Tween-20 (TBS-T) before probing with primary antibodies in 5% BSA-TBS-T overnight at 4°C. Proteins were detected using HRP-conjugated secondary antibodies (Bio-Rad,) and detected using an enhanced chemiluminescence (GE Healthcare) using X-ray film (Fuji) or Alexa Fluor-conjugated secondary antibodies and visualized using ChemiDoc MP (Biorad).

#### Immunofluorescence

Cultured cells were grown to 80% confluence and fixed in 4% PBS-paraformaldehyde for 10 minutes, and incubated in 0.2% Triton X-100 for 10 minutes followed by 5% BSA in PBS-T for 30 minutes. Samples were stained with primary antibody overnight. After 3 washes of PBS-T, secondary antibody staining was performed for 1 h and samples mounted in Vectashield mounting medium containing DAPI (Vector Laboratories).

#### Immunoprecipitation

Cell pellets were washed in ice-cold PBS and suspended in lysis buffer (50 mM Tris [pH 8.0], 150 mM NaCl, 1 mM EDTA, 1% Triton X-100, supplemented with 1 × protease inhibitor and 1 × phosphatase inhibitor cocktails [both Roche], 10 mM sodium butyrate, 10 mM nicotinamide) for 10 min on ice and clarified by centrifugation at 13,000 rpm, 10 min. After retaining an input fraction, samples were incubated overnight with rotation with 1 μg antibody. 50 μl of a 50% protein G-agarose bead slurry (Roche), previously equilibrated in lysis buffer, was added and the samples rotated for a further 2 hours. After extensive washing in lysis buffer, beads were boiled in hot 2 × SDS-PAGE loading buffer. For FLAG IPs, 20 μL of a 50% slurry of anti-FLAG (M2)-conjugated protein G-agarose beads, previously equilibrated in lysis buffer, was directly added to the clarified lysate overnight before washing and boiling.

#### GFP- and HIS-tag pull down

GFP-tagged MITF were purified using GFP-trap (Chromotek Cat#gtma-100) from transiently transfected Phoenix-ampho with indicated plasmids (total 6 μg DNA in 6 cm dish) using Fugene 6. The cells were lysed in 500 μL RIPA supplemented with 5 μM M344 (Stratech Scientific Cat#S2779-SEL) and 4xPIC (Sigma Cat#5892988001) on ice 30 mins before passing through 25 guage needle for mechanical disruption till no visible clump is seen follow by pre-clearing at 14,000 x *g* centrifugation for 10 mins. Supernatant was used for GFP pull-down overnight, wash 3 times and eluted in LDS.

HIS-tag MITF were purified using Ni-NTA His⋅Bind® Superflow Resin (Merck Cat#70691) from 501mel cells stably expressing ectopic MITF-10xHIS. Cells pelleted from 15 cm dish were lysed in 1 mL lysis buffer (6M guanidinium HCl, 0.1 M Na_2_HPO_4_/NaH_2_PO_4_, 10mM Tris-HCl (pH8), 0.005M imidazole, 0.01M β−ME) and briefly sonicated before pre-clearing through centrifugation, 14,000 x *g*, 10 mins. Supernatant is transferred to 15ml LoBind tube with extra 4 mL lysis buffer. 100 μL of washed and pre-equilibrated Ni-NTA resin in lysis buffer was added to the supernatant and rotated overnight, 4°C before washing 1x in lysis buffer, 1x wash buffer (8 M Urea, 0.1 M Na_2_HPO_4_/NaH_2_PO_4_, 10 mM Tris-HCl (pH8), 0.005 M imidazole, 0.01 M β−ME), 2x wash buffer + 0.1% Triton X-100 with 5 min on rotating wheel at room temperature between each wash and eluted in LDS.

#### Mass spectrometry and sample preparation

Cells were transfected with a mammalian Myc-epitope-tagged murine MITF expression vector together with expression vectors for p300. 48 h post-transfection cells were harvested, and whole cell lysate used for immunoprecipitation using anti-Myc antibody. Immunoprecipitates were resolved using SDS-PAGE and gel bands corresponding to MITF were excised, destained then reduced and alkylated and digested with chymotrypsin overnight. Digests were analyzed using a LTQ XL Orbitrap (Thermo, Hemel Hempstead), coupled to a Dionex Ultimate 3000 nano HPLC system (Camberley, Surrey). Data were analyzed using Mascot (Matrixscience, London). Precursor mass tolerance was set to 10 ppm, fragment mass tolerance was 0.5 Da, fixed modification was carbamidomethylation of cysteine and variable modification was oxidised methionine and acetylated lysine. Data were searched against an IPI mouse database.

#### Recombinant protein purification

MITF DNA-binding domains (residues 180-296) that were either WT, K243Q or K243R were expressed in *Escherichia coli* BL21(DE3) cells (Agilent Technologies). Cultures were grown in Luria-Bertani broth to an OD_600_ of 0.7-0.8. Recombinant protein overexpression was induced by addition of isopropyl β-D-1-thiogalactopyranoside (IPTG; 0.5 mM final concentration) and the cultures were incubated for a further 6 h. Cells were harvested by centrifugation, washed in PBS and frozen on dry ice. After thawing, cells were suspended in lysis buffer (50 mM NaH_2_PO_4_, 300 mM NaCl, 10 mM imidazole, 10% v/v glycerol, pH 7.4 [NaOH], 20 mg/ml lysozyme [Invitrogen], 1 × protease inhibitor cocktail [Roche]). Cells were lysed by sonication and centrifuged. Clarified lysate was mixed by rotation with a 50% Ni-NTA slurry (QIAGEN) previously equilibrated in lysis buffer and loaded in gravity flow columns (Bio-Rad). After extensive washing in wash buffer (50 mM NaH_2_PO_4_, 300 mM NaCl, 20 mM imidazole, 10% v/v glycerol, pH 7.4 [NaOH]), bound material was serially eluted in elution buffer (50 mM NaH_2_PO_4_, 300 mM NaCl, 20 mM imidazole, 10% v/v glycerol, pH 7.4 [NaOH]). Fractions were analyzed by SDS-PAGE and Coomassie staining to determine purity, and pure fractions were pooled and glycerol added to a final concentration of 30% v/v.

#### Circular dichroism (CD) spectropolarimetry measurements

Prior to each measurement, samples were dialyzed against 10 mM potassium phosphate pH 7.5, 150 mM NaF and diluted to 0.25 mg x ml^-1^. Spectra were recorded at 10°C on a Chirascan CD Spectrometer (Applied Photophysics), between 185 and 260 nm in a 0.1 cm cuvette. Machine settings were as follows: 1 nm bandwidth, 0.5 s response, and 1 nm data pitch. Spectra were background subtracted and converted to mean residue ellipticity.

#### Fluorescence anisotropy assay

The following fluorescein-labeled oligonucleotides were synthesized at METABION (Planegg/Steinkirchen, Germany):

Consensus: 5′- GAGATCACGTGATGAC-3′-Fluorescein
E-box: 5′- GAGACCACGTGTTGAC-3′-Fluorescein
M-box: 5′- GAGATCATGTGTTGA C −3′-Fluorescein
HS: 5′- GAGATCACGACTTGAC −3′-Fluorescein

These oligonucleotides were annealed with complementary unlabeled oligonucleotides through incubation at 95°C for 5 min, followed by a passive cooling step to room temperature. Increasing concentrations of MITF proteins were incubated with the respective dsDNA oligonucleotides at a final concentration of 1.33 nM at 25°C for 5 minutes in 10 mM Tris/HCl pH 7.5, 300 mM NaCl, 0.01% TRITON-X100, and 0.1 mg/mL BSA. Fluorescence anisotropy was then measured using an Infinite M1000 plate reader (TECAN) using the excitation diode at 470 nm and detecting the emitted light at 530 nm.

#### Electrophoretic mobility shift assays

EMSAs were performed by incubating equal amounts of recombinant proteins diluted in bandshift buffer (25 mM HEPES [pH 7.4], 150 mM KCl, 10% v/v glycerol, 200 μg/ml BSA, 5 μM DTT) for 20 min in the presence or absence of cold competitor oligonucleotides or sonicated salmon sperm DNA (Agilent), prior to addition of ^32^P-labeled oligonucleotide (labeled at both ends with α^32^-P-dCTP, Perkin Elmer) and loading on 6% native acrylamide gels. Gels were dried and visualized by autoradiography. The oligonucleotide sequences used were: TCACGTGA probe (5′-CTAGACTTGTGGAGATCACGTGATGACTTCCTGATTCCT, used for radiolabelling and as cold competitor), and cold competitors: contained the same backbone, but varied in specific bases within the core TCACGTGA motif indicated in the figure. The sequences given plus reverse complement oligonucleotide sequences were from Integrated DNA Technologies, and were annealed before use.

#### Subcellular-fractionation

Cell pellets were re-suspended in 5 pellet volumes of ice-cold nuclear-isolation buffer (10 mM HEPES (pH 7.9), 10 mM KCl, 1.5 mM MgCl_2_, 0.5 mM DTT, 10 mM sodium butyrate, 4 × PIC) and rotated at 4°C for 10 min before NP−40 was added to a final concentration of 0.5% and incubated for a further 5 min. Plasma membrane breakdown was confirmed under the light microscope before centrifugation at 5,000 × *g* for 10 min. The resulting supernatant constituted the cytosolic fraction while the pellet was further resuspended in 3x pellet volumes of nuclear extraction buffer (20 mM HEPES (pH 7.9), 420 mM NaCl, 1.5 mM MgCl_2_, 0.2 mM EDTA, 25% glycerol, 0.5 mM DTT, 10 mM sodium butyrate, 4 × PIC), 4°C, 10 min then centrifuge at 13,000 × *g* for 10 min. The supernatant corresponded to the nucleoplasmic fraction. The insoluble pellet was further resuspended in 3 x pellet volume of nuclear extraction buffer + 10 μl Lysonase (Millipore; Cat# 71230) and incubated 20 min room-temperature followed by centrifuge at 13,000 × *g*,10 min. All four fractions were reconstituted to the same final volume in LDS buffer such that each μl corresponded to the same fraction of cells.

Alternatively, cells were pelleted from a 10 cm dish is resuspended in 500 μl resuspension buffer (20 mM Pipes pH 6.8, 1 mM EGTA pH 6.8, 1 mM MgCl_2_, 1 x PIC) and incubated on ice 5 min before Triton X-100 was added to a final concentration of 0.5%. Plasma membrane breakdown was confirmed under the light microscope before centrifugation at 900 × *g* for 5 min, 4°C. The supernatant corresponds to the cytosolic fraction (Fraction 1). The nuclear pellet was resuspended in 500 μl nuclear extraction lysis buffer (100 mM KCl, 300 mM Sucrose, 10 mM Pipes pH 6.8, 3 mM MgCl_2_, 1 mM EGTA, 1 x PIC and 100 μg/ml DNase) and incubated at 30°C for 45 min with gentle agitation before centrifugation at 1500 × *g* for 5 min at 4°C. The supernatant corresponds to Fraction 2. The pellet was further resuspended in 375 μl nuclear extraction lysis buffer + ammonium sulfate to a final concentration of 0.25 M and incubated for 10 min at RT with gentle agitation before centrifugation at 1500 × *g* for 5 min at RT. The supernatant corresponds to Fraction 3. The pellet was then resuspended in 300 μl ice-cold nuclear extraction lysis buffer plus NaCl to a final concentration of 2 M and incubated on ice for 10 min before centrifugation at 4,000 × *g* for 5 min at 4°C. The supernatant corresponds to Fraction 4 while the pellet corresponds to insoluble nuclear matrix protein, Fraction 5.

#### ChIP-seq

Cells from three 80%confluent 15 cm dishes were trypsinised, collected into a 50 ml falcon tube (Corning; Cat# 430828), centrifuged (800×g,4min) and media aspirated. Cross-linking was done by adding 45 ml ice-cold PBS containing 0.4% paraformaldehyde. Cells were rotated 10 min at RT before quenching with glycine to a final concentration of 0.2 M for10min. Samples were then washed and centrifuged (1500×g,10min). A total of thirty 15 cm dishes were used for each replicate of ChIP-seq. Lysis was done in 1 ml ChIP lysis buffer (50 mM Tris-HCl (pH8.0), 10 mM EDTA, 10 mM sodium butyrate,1%SDS, 4×PIC (Roche; Cat#05056489001)) by passing the cell suspension through a 25 guage needle until there were no visible clumps before sonicated for approximately 12 min in a Covaris S220 (Peak incident = 145 W, Duty Factor = 8%, Cycle/Burst = 200) until 200−400 bp fragments were obtained (assessed by Bioanalyzer using Agilent High Sensitivity DNA Kit (Agilent; Cat# 5067-4626). The sonicated chromatin was cleared by centrifugation at13,000×g for 10 min and the supernatant diluted 8-fold in ChIP dilution buffer (16.7 mM Tris (pH8.0), 167 mM NaCl, 1.2 mM EDTA, 1% Triton X−100, 0.01% SDS) before 120 μg of anti-HA antibody (Roche; Cat# 11666606001) was added and chromatin rotated in a 50 ml falcon tube overnight. In parallel 550 μl Dynabeads G were washed, resuspended in ChIP dilution buffer, and blocked in 0.5 mg/ml BSA overnight. Immunoprecipitation was carried out using blocked-Dynabeads, rotated for 1 hr, 4°C. The beads were washed three times each in low salt wash buffer (20 mM Tris-HCl (pH8.0), 150 mM NaCl, 2 mM EDTA, 1% Triton X−100,0.1%SDS), high salt wash buffer (20 mM Tris-HCl (pH8.0), 500 mM NaCl, 2 mM EDTA,1%Triton X−100,0.1%SDS) and LiCl wash buffer (10 mM  Tris-HCl (pH8.0), 250 mM LiCl, 1 mM EDTA, 1%sodium deoxycholate, 1%NP−40) with beads transferred to a new DNA LoBind tube (Eppendorf; Cat# Z666548) with each wash. The beads were eluted in 0.2 ml elution buffer (100 mM NaHCO_3_,1%SDS). Reverse cross-linking of ChIPed-DNA was done at 55°C overnight with addition of 0.3 M NaCl (final concentration), 20 μg RNase A (Invitrogen; Cat# 12091021) and 20 μg  Proteinase K (Roche; Cat# 3115828001). Recovery of ChIPed-DNA was done using QIAquick PCR Purification Kit (QIAGEN; Cat# 28106). The concentration of ChIPped-DNA was assessed using Qubit dsDNA HS Assay Kit (Invitrogen; Cat# Q32851).

Alternately, cells were lysed in ChIP lysis buffer and chromatin sheared to around 300 bp for 16 minutes in a Covaris S220 at 140 W Peak Incident Power, 5% Duty Cycle and 200 Cycles per Burst. 70 μg chromatin was diluted 9 times in ChIP dilution buffer and pre-cleared for 2 hours at 4°C with 50 μL of 50% protein-G Sepharose slurry (Roche), previously blocked with 0.5 mg/ml BSA and 0.5 mg/ml sonicated salmon sperm DNA. The supernatant was incubated overnight with 5 μg of anti-HA antibody (clone 12CA5, Roche) before 50 μL pre-blocked protein-G Sepharose slurry was added for 1 hour. Beads were washed two times each in low salt buffer, high salt buffer, LiCl buffer and TE buffer (10 mM Tris-HCl, 1 mM EDTA, pH 8.0). Chromatin was eluted in elution buffer, cross-links were reversed overnight at 65°C with 0.3 M NaCl and 10 μg RNase A followed by addition of 4 mM Tris-HCl (pH 8.0), 10 mM EDTA (pH 8.0) and 20 μg Proteinase K (Fermentas) for 1 hour at 42°C, and DNA purified with phenol-chloroform before ethanol precipitation with 1 μg glycogen [Roche]. Samples which showed enrichment at expected targets on qPCR (as described in Louphrasitthiphol et al., 2019) were subjected to sequencing using a HiSeq4000(Illumina) at the Wellcome Trust genomic service.

##### Bioinformatic analysis

All fastq files were quality checked with FastQC. Reads from steady state experiments were aligned to human genome build hg19 (GRCh37v75) using Bowtie 1.1.2 (Langmead and Salzberg, 2012), allowing for 1 mismatch. Duplicate reads were discarded using PicardTools version 1.96, http://picard.sourceforge.net. Library-count normalized read density was examined at specific loci using the University of Santa Cruz (UCSC) Genome Browser. Read density clustering of normalized libraries was performed with seqMINER (Ye et al., 2011) and heatmaps drawn with deepTools (Ramírez et al., 2014); the regions within each cluster were further sorted by maximal WT MITF read density. Average read density profiles were generated with BedTools. Peaks were identified using MACS2 v2.1.0 (Zhang et al., 2008) and associated with a gene if they fell within 20 kb of the gene body of a RefSeq gene using CisGenome (Ji et al., 2008). *De novo* consensus motifs were predicted with MEME (Machanick and Bailey, 2011) by interrogating the 60 bp around the summits of peaks. The coordinates of all CATGTG and CACGTG motifs were determined genome-wide and overlapped with peak coordinates using BedTools (Quinlan and Hall, 2010). Melanoma patient H3K27ac ChIP-Seq from GEO series GSE6066 (Verfaillie et al., 2015) and bigwig files analyzed as above. Melanocyte MITF ChIP-Seq from GSE50681 (Webster et al., 2014) was processed as above. All additional steps were performed in R (Sato et al., 1997).

To exclude bioinformatics pipeline-specific artifacts, inducible MITF ChIP-Seq samples were processed in two independent ways with similar results obtained with both methods. First: alignment to human genome build hg38 (GRCh38v23) with STARv 2.5.1b (Dobin et al., 2013); downstream analysis including peak calling and annotation, gene ontology analysis, bedgraph generation and *de novo* motif identification using the exact peak coordinates conducted with Homer (Heinz et al., 2010); histograms of read density were visualized using TreeView (Saldanha, 2004). Second: reads were aligned to human genome build hg19 (GRCh37v75) with BWAv0.7.8 and duplicate reads marked with PicardTools; peaks were called with MACS2 using a minimum FDR of 0.001 and annotated with Homer; input-corrected bigwig files were generated with deepTools motif analysis was carried out with BedTools; consensus motifs were identified with MEME using a 60 bp sequence centered on the peak summit.

#### Single molecule tracking

Cells stably expressing HaloTag-MITF (WT or mutant) under the control of a doxycycline inducible promoter were plated in 4-well Nunc Labtek Chambers (Thermo-Fisher, Milan, Italy). 24 h after induction with 20 ng/ml of doxycycline, HaloTag-MITF was labeled by adding 100 pM of the cell-permeable HaloTag ligand Janelia Fluor® 549 (Janelia Farm, Ashburn, VA, USA) to each of the wells. The cells were incubated with the dye for 30’ and then extensively washed with PBS (3 washes), followed by an incubation step (20’ at 37°C in cell culture medium) and one last wash with PBS, and one with cell culture medium. The acquisition of single molecule movies were performed on a previously described custom-made HiLo microscope (Loffreda et al., 2017), equipped with a temperature and CO_2_ Control (set to 37°C and 5% respectively). For the estimation of diffusion coefficients and bound-fractions, we used a laser exposure of 2 ms and an acquisition frame rate 100 fps and collected 1000 frames/movie. Fluorescence was excited with a 561 nm solid-state laser (IFlex Mustang, QiOptiq) and with a laser power density of approximately 1.2 kW/cm^2^. Movies were tracked and analyzed using our previously described software developed in MATLAB (Loffreda et al., 2017, Mazza et al., 2013). Briefly, the resulting tracks are used to populate the histogram of the distribution of displacements (with a bin size Δr = 20nm), that is then fit with a three-component diffusion model, that gives the probability of observing a displacement in the interval [r−(Δr/2),r+(Δr/2)]

$$p\left(r\right)=r\text{Δ}r\sum_{i=1}^{3}\frac{F_{i}}{2D_{i}\text{Δ}t}\exp\left(-\frac{r^{2}}{4D_{i}\text{Δ}t}\right)$$

Where Δt is the interval between two acquisitions (10 ms) and $F_{i}$ is the fraction of molecules with diffusion coefficient equal to $D_{i}$. To provide standard deviations on the fitting parameters a jackknife procedure was adopted as described in Elf et al. (2007) and Loffreda et al. (2017). Briefly, we performed multiple fitting iterations, each of them after dropping 20% of the data for each of the dataset. Errors are provided as standard deviations of the obtained distribution of parameters following 200 individual fitting iterations. The diffusion coefficient of the slowest diffusing component $D_{1}=0.07\left(\text{μ}{\text{m}}^{2}/\text{s}\right)$, compatible with the diffusion coefficients of chromatin bound proteins (Chen et al., 2014), and $F_{1}$ therefore represent the fraction of molecules detected as immobilized to chromatin (as further validated by analyzing the MITF-Δbasic mutant). The MITF-Δbasic mutant also result in the increase of the diffusion coefficients of the unbound population, $D_{2}$ and $D_{3}$. In previous work this kind of observation has been interpreted as the presence of transient binding (at a time-scale faster than the acquisition rate) slowing down diffusion of the WT protein, named effective diffusion (Elf et al., 2007, Sprague et al., 2004). In our case, the free populations of the WT MITF and K243 mutants are all similarly slowed down compared to MITF-Δbasic (Figure 6E), indicating that transient non-specific binding affects all these three forms of MITF. We can calculate the number of detectable long-lived binding events (longer than Δt) occurring in a cell at any given time $\left(N_{s}\right)$, by knowing the number of MITF molecules $N_{MITF}$, as: $N_{s}=N_{MITF}\cdot F_{1}$. Here for $N_{MITF}$ we have used MITF 250,000 dimers estimated by comparing the level of MITF detected in a western blot from 501mel cells compared to that detected using the same anti-MITF antibody of a dilution series of a known concentration of bacterially expressed and purified MITF. Similarly, in the case of effective diffusion the number of transient binding events $N_{ns}\;$can be estimated by comparing the diffusion coefficients of the free populations of WT MITF and K243 mutants to the diffusion coefficients of the MITF-Δbasic mutant, as Elf et al. (2007) and Sprague et al. (2004):

$$N_{ns}=N_{Mitf}\left(1-F_{1}\right)1-\frac{D_{free,MITF}}{D_{free,\;\text{Δ}basic}}$$

Where, $D_{free,\;MITF}$ refers to the diffusion coefficients of the free population of the one of MITF proteins (WT or K243 mutants) and $D_{free,\text{Δ}basic}$ refers to the diffusion coefficients of the free population of the MITF-Δbasic mutant. As two diffusion coefficients ($D_{2}$and $D_{3}$) are estimated for the free populations of each of the protein we provide $N_{ns}\;$ as a range, where the two limits of the interval are calculated using $\left(D_{2,MITF}/D_{2,\;\text{Δ}basic}\right)$ and $\left(D_{1,\;MITF}/D_{1,\text{Δ}basic}\right)$ respectively. The residence time of this transient binding events cannot be measured, as it is faster than the acquisition frame rate.

For the measurement of MITF residence times at more stable binding sites (those resulting in the immobile fraction described above), we scaled down the laser power by a factor 20, and collected movies using long exposures (200 ms exposures, frame rate 2 Hz), in order selectively image immobilized molecules due to motion blur of diffusing MITF (Chen et al., 2014). The complement cumulative distribution of the duration of binding events 1−CDF(t) were fitted a two-component exponential decay (Chen et al., 2014, Loffreda et al., 2017):

$$1-CDF\left(t\right)=f_{1}\;\exp\left(-\frac{t}{\tau_{1}}\right)+\left(1-f_{1}\right)\exp\left(-\frac{t}{\tau_{2}}\right)$$

To extract the characteristic intermediate-lived $\tau_{1}\;$and long-lived$\;\tau_{2}$ binding times to sites and the fraction of binding events $f_{1}$ and $1-f_{1}$ occurring at these sites. The average residence time is then calculated as:

$$<\tau >\;=f_{1}\tau_{1}+\left(1-f_{1}\right)\tau_{2}$$

### Quantification and Statistical Analysis

Figure 1B. Top 900 significant peaks defined top 900 ranked peaks from MACS2 v2.1.0 output against background control. q-value cutoff = 0.05
Figure 1F. Boxplots show the interquartile range (IQR), with the median value marked by the line transecting the box. Values falling outside the IQR but within the range of IQR ± 1.5 × IQR are shown by the dashed line whisker. Where included, the notch indicates the 95% confidence interval about the median, and further outliers represented by dots.
Figure 3C. Binding data were analyzed using the GraphPad Prism software. Binding profiles were fitted using a simple model assuming a stoichiometry of one MITF dimer per double stranded DNA fragment. When K_D_ values were higher than 500 nM, the maximum specific binding value (Bmax) was estimated according to the one obtained from other variants and maintained fixed for the fitting. K_D_ values reported correspond to the means of three independent measurements and the ± error numbers represent the standard deviations.
Figure 4A. *p-value*s were calculated using a Two-Sample *t*-test, and the line in the box and whisker plots indicates the mean. The exact number of embryos injected and melanocytes count are shown in Figure S3.
Figure 4E. *p-value*s determined using Student’s *t*-test when comparing WT and K243Q mutant is ≤ 0.003. Error-bar indicated SEM.
Figure 5A. Center of the peaks were defined as the summit of each individual peak. The number of row = 103911, derived from WT 20 ng reference.
Figure 5B.
All peaks were called using the following fixed parameters
# FDR rate threshold = 0.001000000
# Fold over input = 4.00
# Poisson p value over input = 1.00e-04
# Fold over local region = 4.00
# Poisson *p-value* over local region = 1.00e-04
and variable parameters (modeled according to each dataset as these are sequencing depth dependent)
# FDR effective poisson threshold
# FDR tag threshold
Figure 5E. Boxplots show IQR with median value marked by line of the same color as the box and mean value marked by black line. Whiskers cover values between IQR and ± 1.5 × IQR. Any values outside 1.5 × IQR are plotted with circle.
Figures 6C–6F.$\;N_{cells}=20,\;6,\;15,\;15;\;N_{displacements}=17802,\;2684,\;16422,\;12999$ for MITF WT, Δbasic, K243Q, K243R respectively. Error bars indicate SD.

## Supplemental Information

Document S1. Figures S1–S6 and Table S2

Table S1. MITF-Bound Genes Stratified by Peak Height, Related to Figure 1

Document S2. Article plus Supplemental Information
