# Supplementary material for: Tuning Transcription Factor Availability through Acetylation-Mediated Genomic Redistribution
Source: Mol Cell. 2020 Aug 6;79(3):472–487.e10. doi: 10.1016/j.molcel.2020.05.025 (PMC7427332; doi:10.1016/j.molcel.2020.05.025)
Supplement: Document S1. Figures S1–S6 and Table S2 [file mmc1.pdf]

Molecular Cell, Volume *79*

Supplemental Information

Tuning Transcription Factor Availability through Acetylation-Mediated Genomic Redistribution

Pakavarin Louphrasitthiphol, Robert Siddaway, Alessia Loffreda, Vivian Pogenberg, Hans Friedrichsen, Alexander Schepsky, Zhiqiang Zeng, Min Lu, Thomas Strub, Rasmus Freter, Richard Lisle, Eda Suer, Benjamin Thomas, Benjamin Schuster-Böckler, Panagis Filippakopoulos, Mark Middleton, Xin Lu, E. Elizabeth Patton, Irwin Davidson, Jean-Philippe Lambert, Matthias Wilmanns, Eiríkur Steingrímsson, Davide Mazza, and Colin R. Goding

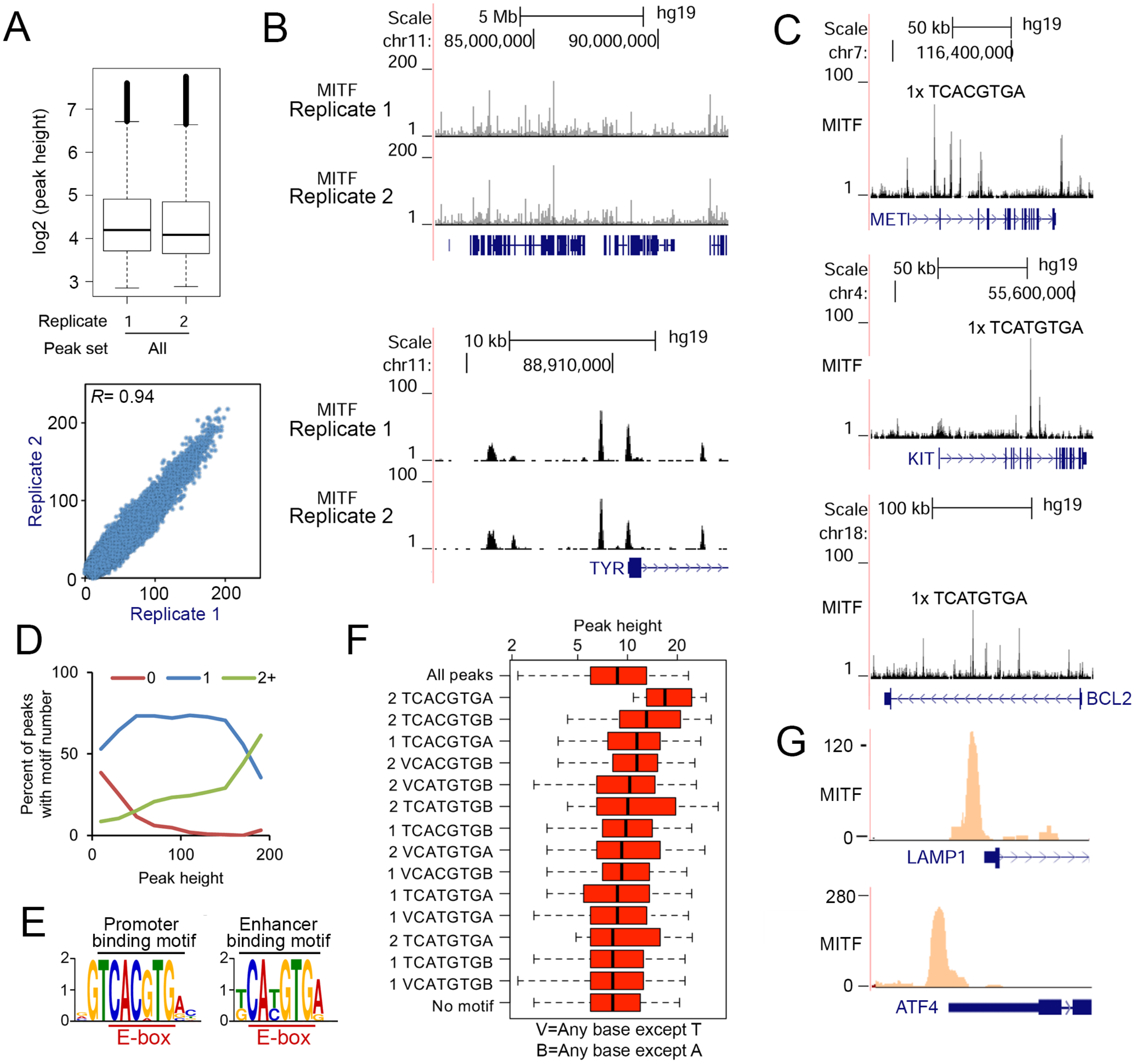

Supplemental Figure S1 Related to Figure 1

MITF ChIP-seq analysis

(A) Boxplots of log2 of peak heights from two replicate 501mel HA-MITF ChIP-Seq experiments (top) and inter-replicate correlation (bottom).

(B) Genome browser screenshot of HA-MITF ChIP-Seq in the indicated region of chromosome 11, as well as a zoomed in view of the 5'-end of *Tyrosinase*.

(C) Genome browser screenshots of HA-MITF binding associated with *MET*, *KIT* and *BCL2*.

(D) Peaks were divided into equal width bins based on height, and the percent of peaks within each bin containing the indicated number of 5'-TCA[T/C]GTGN-3' motifs calculated.

(E) Consensus MITF binding sites predicted from 60 bp regions around peak summits in promoters or enhancers generated by MEME from ChIP-seq using anti-MITF antibody in melanocytes using dataset GSE50681.

(F) Box and whisker plots derived from ChIP-seq of MITF in melanocytes (GSE50681) relating peak height to sequence beneath the peak.

(G) Genome browser screenshots showing binding of endogenous MITF to the ATF4 and LAMP1 loci.

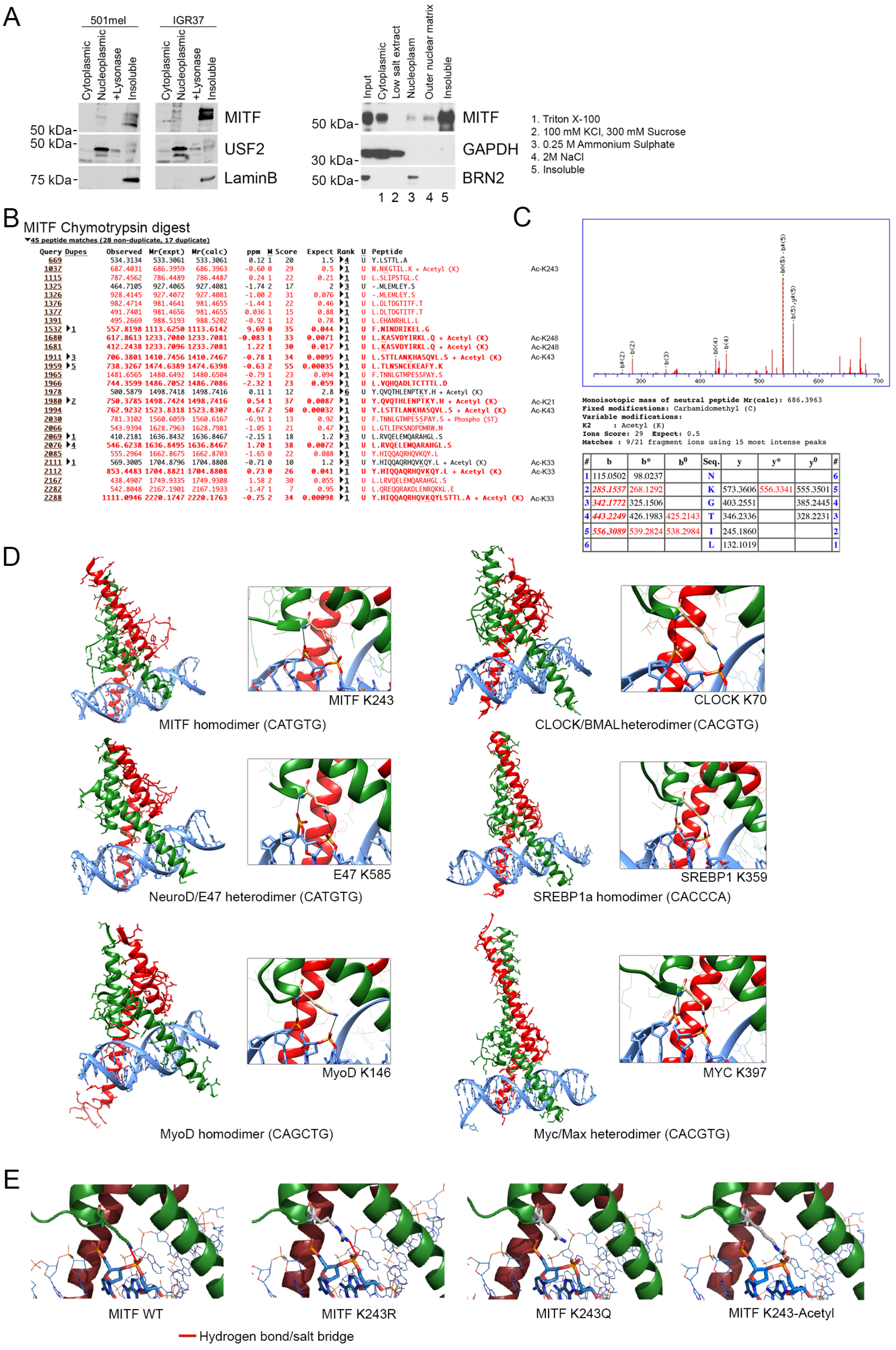

Supplemental Figure 2. Related to Figure 2

MITF and other bHLH(LZ) factors are acetylated on K243 or equivalent residues

(A) Western blotting using indicated antibodies of fractions derived from indicated cell lines. Extraction conditions are described in detail in the Methods. BRN2 and USF2 were used as controls for MITF.

(B,C) Mass spectrometry results of Myc-epitope tagged MITF expressed in HEK293 cells together with p300 and immunoprecipitated using anti-Myc antibody before chymotrypsin digestion and analysis.

(D) DNA co-crystal structures of indicated bHLH and bLHL-LZ factors showing conservation of MITF K243 equivalent residues making DNA phosphate backbone contact.

(E) Modeling the interaction between K243 WT and mutants and the DNA phosphate backbone. Assuming the mutations cause no major structural changes as observed in the circular dichroism data (Figure 3A), residue side chains were simply mutated on the 3D structure of MITF/M-box in Pymol, and acetyl-lysines were built with the help of the PyTMs plug-in. Snapshots of representative conformations of variant amino acids 243 are depicted as in the Figure 2F close-up. They show that R243, although bulkier, maintains the ability to form strong hydrogen bond to the DNA backbone. In contrast Q243 harbors a shorter, charge neutral and polar side chain. Acetyl-K243 (Ac-K243) exhibits a chemically similar side chain to Q243 and although potentially prone to steric clashes in the area, the flexibility of the loop makes this uncertain.

MITF mutation	Number of embryos injected	Number of embryos with 1+ melanocytes	Average number of melanocytes	*p*-value
WT	73	59 (80.8%)	12.4	
K238Q	85	68 (80.0%)	10.7	K238Q vs WT : 0.34
K238R	124	43 (34.7%)	2.2	K238R vs WT: 6.07E-08<br>K238R vs K238Q: 6.79E-15
Uninjected	43	0	0	

Supplemental Figure 3 Related to Figure 2

Statistical analysis of nacre zebrafish injected with the indicated WT and K238 (K243 equivalent) MITF mutants related to Figure 4A. *P*-values were generated using a Two-Sample T-Test.

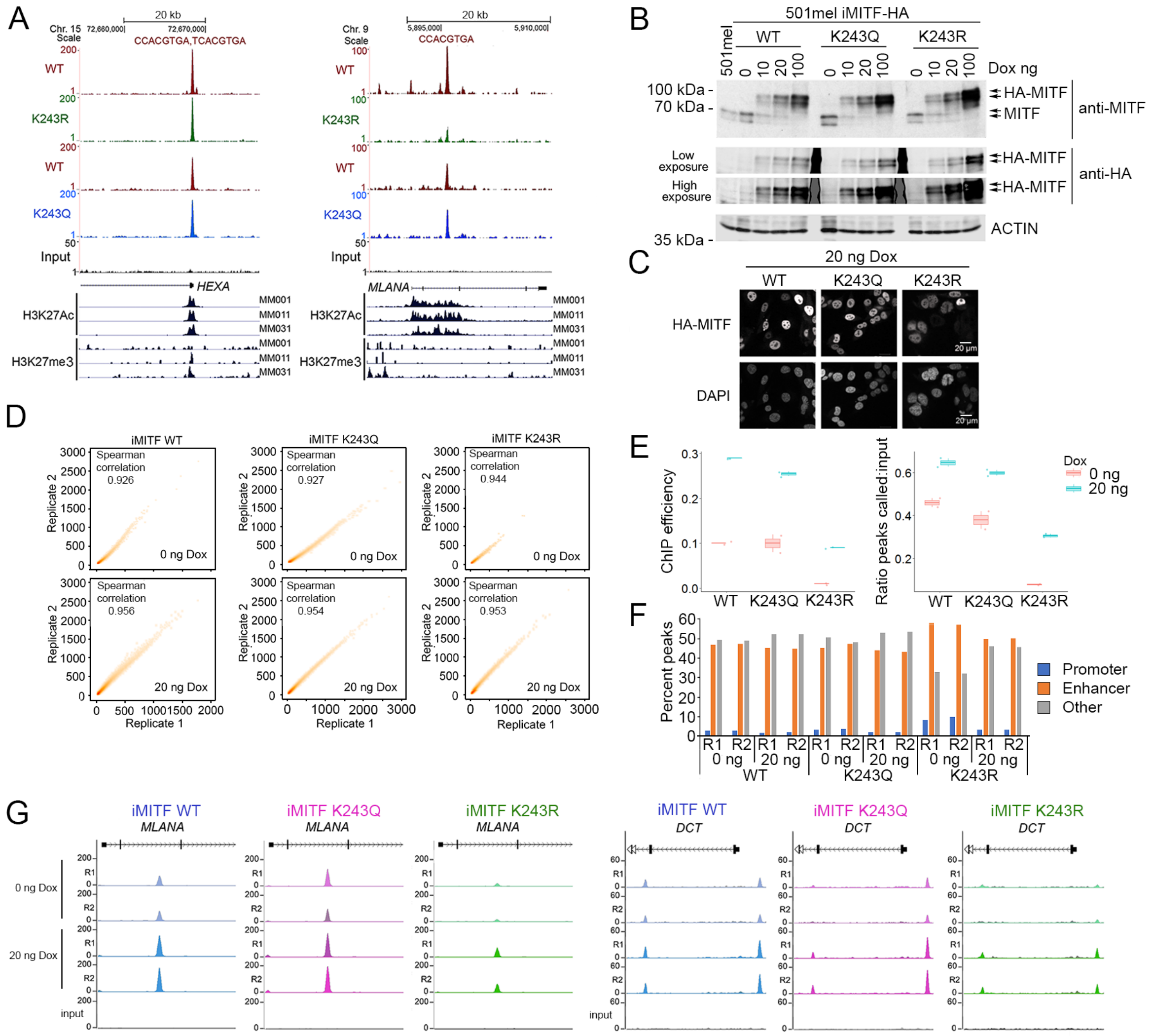

Supplemental Figure 4 Related to Figure 5

(A) UCSC browser screenshots of ChIP-seq using indicated HA-tagged MITF WT and mutants expressed constitutively in 501mel cells as shown in Figure 4B. Indicated below are the ChIP-seq profiles of H3K27me3 and H3K27Ac derived from human melanomas described in Verfaillie et al (2015).

(B) Western blots of 501mel cell lines inducibly expressing indicated HA-tagged MITF WT and K243 mutants. Cells were induced with increasing concentration of doxycycline and western blotted using indicated antibodies. MITF runs as two bands corresponding to differentially phosphorylated forms. Using anti-MITF antibody, endogenous MITF is decreased as ectopic HA-tagged MITF expression increases.

(C) Immunofluorescence using anti-HA antibody to detect ectopically expressed MITF WT and mutants in inducible cell lines following induction with 20 ng doxycycline. Scale bar indicates 20 µm.

(D) Reproducibility of duplicate ChIP-seq experiements using indicated HA-MITF WT and K243 mutants expressed after treatment of cells with 0 or 20 ng doxycycline. Numbers indicate peak score/height.

(E) Box and whisker plots showing ChIP-efficiency or Ratio of peaks called/input as indicated using WT or K243 mutants expressed using 0 or 20 ng doxycycline.

(F) Relative distribution of ChIP-seq peaks for WT and K243 mutant MITF between enhancers, promoters and other sites after treatment of 501mel cells with 0 or 20 ng doxycycline.

(G) UCSC genome browser screenshots showing HA-MITF WT and K243 mutant binding at indicated loci after induction with 0 or 20 ng doxycycline.

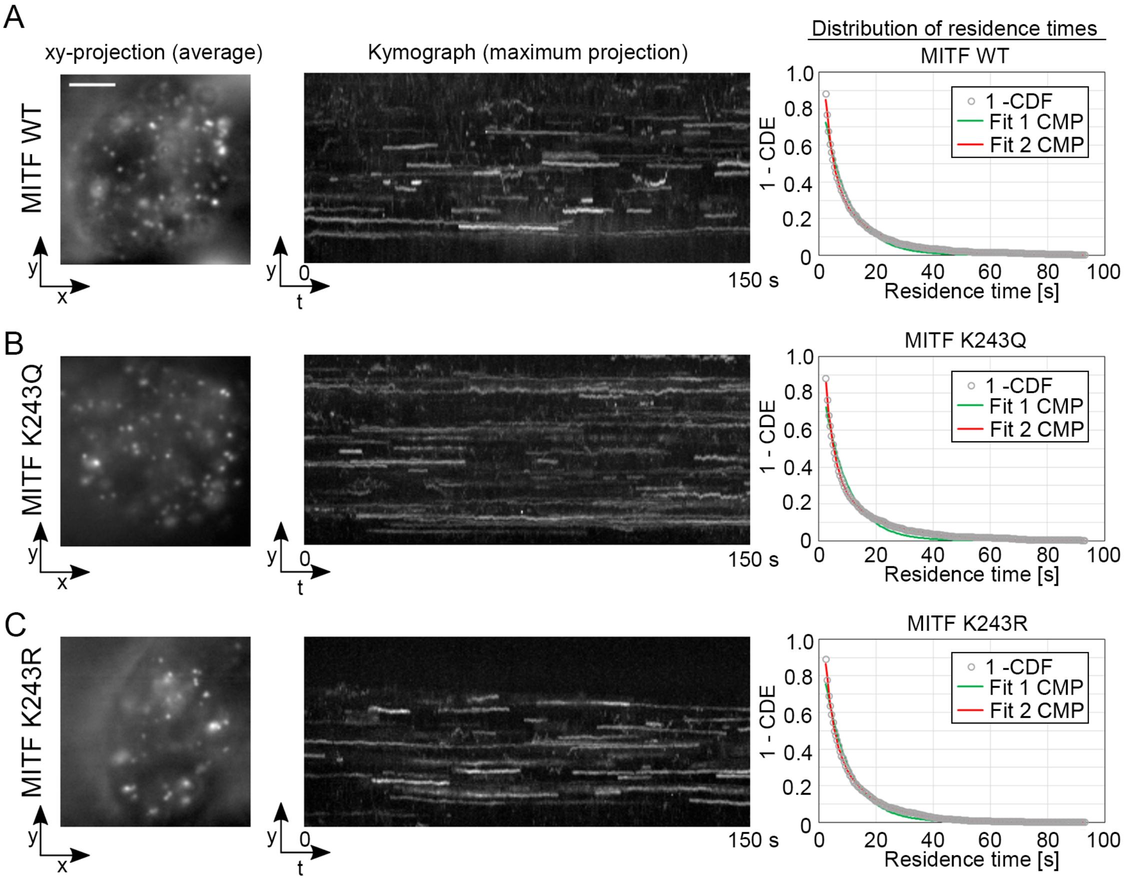

D

	WT	K243Q	K243R
Fully free fraction	26 ± 2 %	37 ± 3 %	43 ± 7 %
Short lived bound fraction	30 ± 2 %	28 ± 3 %	40 ± 7 %
Intermediate bound fraction	30 ± 2 %	26.8 ± 1.4 %	11.2 ± 0.8 %
Long lived bound fraction	13 ± 2 %	8.0 ± 1.5 %	6 ± 0.8 %
Short lived residence time	n.d. (<10 ms)	n.d. (<10 ms)	n.d. (<10 ms)
Intermediate residence time	3.04 ± 0.19 s	3.17 ± 0.07 s	2.91 ± 0.13 s
Long lived residence time	15.3 ± 0.6 s	15.3 ± 0.3 s	13.4 ± 0.3 s

Supplemental Figure 5 Related to Figure 6

Long-exposure single-molecule tracking for measurement of HaloTag-MITF residence times on chromatin. Displayed are the average projections, the kymographs and the distribution of residence times for (A) MITF WT, (B) MITF K243Q, (C) MITF K243R mutant. (D) Results of fitting the distributions of residence times.

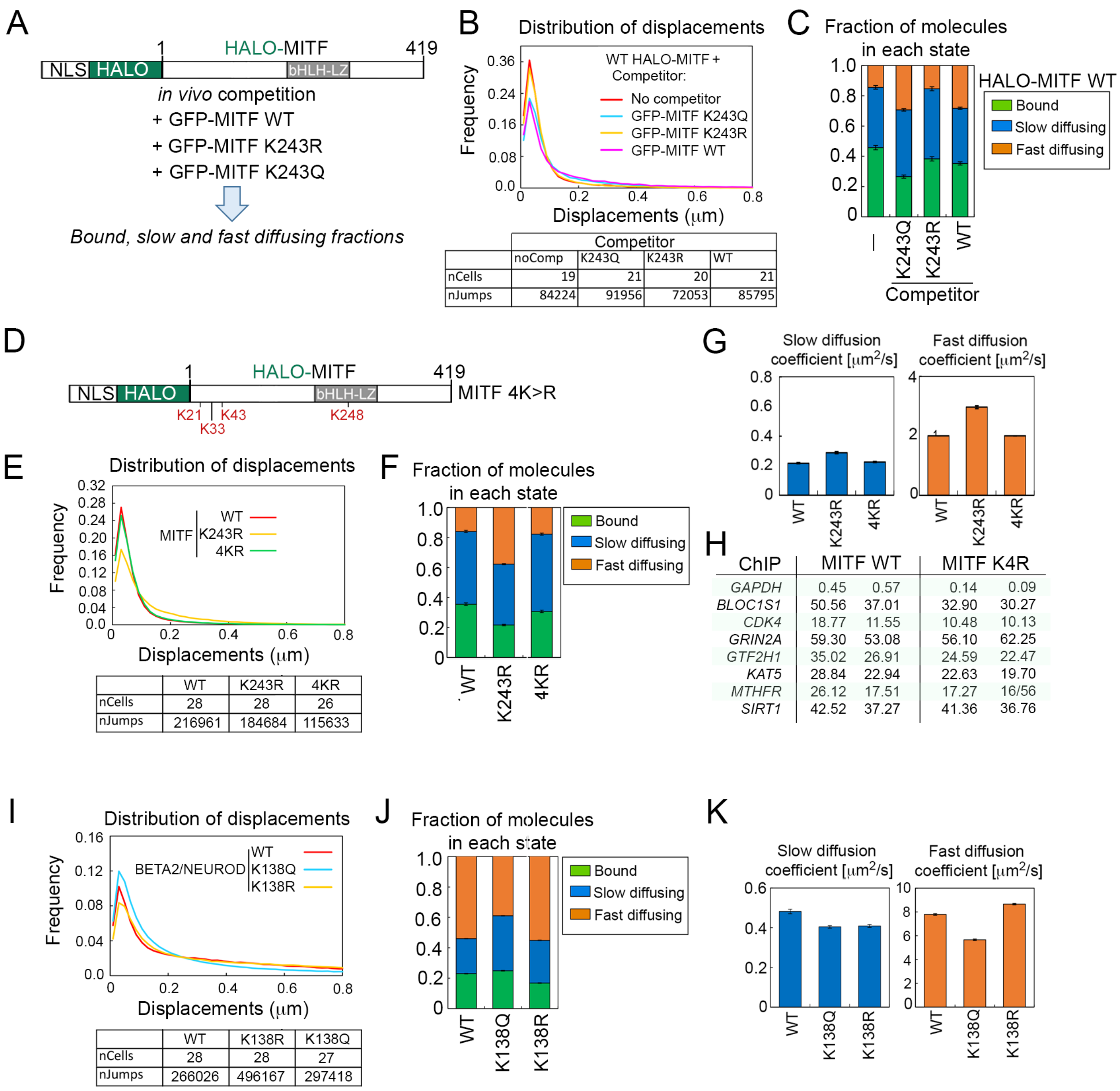

Supplemental Figure S6 related to Figure 5

(A) Schematic of SMT competition assay.

(B) Distribution of SMT displacements of HALO-MITF WT expressed alone or after doxycycline-mediated induction of GFP-MITF WT or K243Q or K243Q mutants as competitors. The SMT movies were tracked to generate the distribution of single-molecule displacements between consecutive frames that was next fit with a three-component model (one immobile component and two diffusing components), to provide quantitative estimates for WT MITF and indicated mutants shown in (C). The numbers of cells and events measured is shown below.

(C) Results of SMT competition assay showing bound, fast and slow diffusing fractions of cells expressing HALO-WT MITF alone, or after doxycycline-mediated induction of GFP-WT MITF or K243Q or K243QR mutants. Error bars indicate SD.

(D) Schematic showing expression vector for the HALO-MITF 4KR mutant

(E) Distribution of SMT displacements of HALO-MITF WT and K243R and K4R mutants. Data were analysed as in (B) to provide quantitative estimates for WT MITF and indicated mutants shown in (F) and (G). The numbers of cells and events measured is shown below.

(F) Quantitative estimates derived from SMT using WT and mutant HALO-tagged MITF for the fraction of molecules in each indicated state. Error bars indicate SD.

(G) Quantitative estimates of the diffusion coefficients of free molecules. For MITF WT, K243R, and 4KR mutant respectively. Error bars indicate SD.

(H) ChIP-qPCR of MITF WT and 4KR mutant at indicated genes. Results of biological duplicates are shown.

(I) Distribution of SMT displacements of HALO-BETA2/NEUROD WT and K138R and K138Q analysed as in (B). The numbers of cells and events measured is shown below.

(J) Quantitative estimates derived from SMT using WT and mutant HALO-tagged BETA2/NEUROD for the fraction of molecules in each indicated state. Error bars indicate SD

(K) Quantitative estimates of the diffusion coefficients of free molecules. For BETA2/NEUROD WT, K138R, and K138Q mutant respectively. Error bars indicate SD.

Mitf	Dox	Rep	FDR effective poisson threshold	FDR tag threshold	Fragment length	Peaks called	Differential peaks (passed)	Local background filtering (passed)	Clonal filtering (passed)
**WT**	0ng	R1	8.41E-06	26	220	119608	57506	50246	50246
**WT**	0ng	R2	1.15E-05	27	220	138626	60813	52780	52780
**WT**	20ng	R1	1.46E-05	28	236	155946	103772	94981	94981
**WT**	20ng	R2	9.41E-06	28	222	173177	108611	99512	99512
**K243Q**	0ng	R1	9.91E-06	29	210	153799	64651	57516	57516
**K243Q**	0ng	R2	1.30E-05	28	190	152505	51403	44031	44030
**K243Q**	20ng	R1	1.63E-05	27	222	173712	101643	94582	94582
**K243Q**	20ng	R2	1.12E-05	26	222	173492	106095	99342	99342
**K243R**	0ng	R1	6.00E-06	27	204	121255	9299	6770	6770
**K243R**	0ng	R2	6.34E-06	24	194	104860	8506	6212	212
**K243R**	20ng	R1	1.23E-05	26	204	131090	41209	37227	37227
**K243R**	20ng	R2	8.91E-06	29	220	143926	42817	38462	38462

SUPPLEMENTAL TABLE 2. **Quantification and statistical analysis corresponding to Figure 5B**

SUPPLEMENTAL INFORMATION QUANTIFICATION AND STATISTICAL ANALYSIS

Supplemental Figure 1A and 1F

Boxplots show the interquartile range (IQR), with the median value marked by the line transecting the box. Values falling outside the IQR but within the range of IQR ± 1.5×IQR are shown by the dashed line whisker. Where included, further outliers are represented by dots.

Supplemental Figure 1A bottom – Pearson correlation value

Supplemental Figure 4D Inter-replicate ρ values were calculated using Spearman rank correlation

Supplemental Figure 4E

Samples	ChIP_efficiency	Group	Dox	Ratio_Peaks_called_over_Input
iMitf_WT_0ng_R1	0.10	WT	0 ng	0.480787238
iMitf_WT_0ng_R2	0.10	WT	0 ng	0.438683941
iMitf_WT_20ng_R1	0.29	WT	20 ng	0.665435471
iMitf_WT_20ng_R2	0.29	WT	20 ng	0.62716758
iMitf_K243Q_0ng_R1	0.12	K243Q	0 ng	0.42036034
iMitf_K243Q_0ng_R2	0.08	K243Q	0 ng	0.337057801
iMitf_K243Q_20ng_R1	0.25	K243Q	20 ng	0.585123653
iMitf_K243Q_20ng_R2	0.26	K243Q	20 ng	0.611526756
iMitf_K243R_0ng_R1	0.01	K243R	0 ng	0.076689621
iMitf_K243R_0ng_R2	0.01	K243R	0 ng	0.081117681
iMitf_K243R_20ng_R1	0.09	K243R	20 ng	0.314356549
iMitf_K243R_20ng_R2	0.09	K243R	20 ng	0.297493156

Supplemental Figure 4F

MITF	Dox	Rep	Peak counts				Percent peaks		
			Peaks	Promoter	Enhancer	Other	Promoter	Enhancer	Other
WT	0 ng	R1	49513	1530	23297	24686	3.09	47.05	49.86
		R2	52085	1653	24773	25659	3.17	47.56	49.26
	20 ng	R1	102673	2227	46605	53841	2.17	45.39	52.44
		R2	109628	2496	49678	57454	2.28	45.32	52.41
K243Q	0 ng	R1	66113	2285	30118	33710	3.46	45.56	50.99
		R2	50631	2089	24072	24470	4.13	47.54	48.33
	20 ng	R1	101512	2479	44943	54090	2.44	44.27	53.28
		R2	102778	2462	44899	55417	2.4	43.69	53.92
K243R	0 ng	R1	6850	600	3985	2265	8.76	58.18	33.07
		R2	5252	541	3011	1700	10.3	57.33	32.37
	20 ng	R1	39048	1412	19603	18033	3.62	50.2	46.18
		R2	42528	1505	21403	19620	3.54	50.33	46.13

Supplemental Figure 6 The numbers of cells and displacement are indicated in the figure. Error bars indicate SD.
